# Impact of model calibration on cost-effectiveness analysis of cervical cancer prevention

**DOI:** 10.1038/s41598-017-17215-2

**Published:** 2017-12-08

**Authors:** David Moriña, Silvia de Sanjosé, Mireia Diaz

**Affiliations:** 1 Unit of Infections and Cancer - Information and Interventions (UNIC - I&I), Cancer Epidemiology Research Program (CERP), Catalan Institute of Oncology (ICO)-IDIBELL, L’Hospitalet de Llobregat, Barcelona, Spain; 2 Cancer Epidemiology Research Program (CERP), Catalan Institute of Oncology (ICO)-IDIBELL, L’Hospitalet de Llobregat, Barcelona, Spain; 3 Centro de Investigación Biomédica en Red (CIBERESP), Barcelona, Spain; 4 Centro de Investigación Biomédica en Red (CIBERONC), Barcelona, Spain

## Abstract

Markov chain models are commonly used to simulate the natural history of human papillomavirus infection and subsequent cervical lesions with the aim of predicting future benefits of health interventions. Developing and calibrating these models entails making a number of critical decisions that will influence the ability of the model to reflect real conditions and predict future situations. Accuracy of selected inputs and calibration procedures are two of the crucial aspects for model performance and understanding their influence is essential, especially when involves policy decisions. The aim of this work is to assess the health and economic impact on cervical cancer prevention strategies currently under discussion according to the most common methods of model calibration combined with different accuracy degree of initial inputs. Model results show large differences on the goodness of fit and cost-effectiveness outcomes depending on the calibration approach used, and these variations may affect health policy decisions. Our findings strengthen the importance of obtaining good calibrated probability matrices to get reliable health and cost outcomes, and are directly generalizable to any cost-effectiveness analysis based on Markov chain models.

## Introduction

Cost-effectiveness analysis popularity is growing in the last years as an approach for guiding the efficient allocation of scarce health care resources and has a large impact on the development, implementation, and evaluation of health policies. A better understanding of model performance is important to assess the impact on health outcomes and ensuing policy decisions. Most analyses involve the use of mathematical models to disease simulation that synthesize data from various sources. These models are imbued with an appreciable degree of uncertainty, at methodological, modeling or structural, and parameterisations level^1^. Some guidelines on health economic evaluations consider pivotal to assess the implications of uncertainty in the results, either through statistical analysis or through sensitivity analysis^2,3^. Much has been written in how to handle uncertainty using deterministic and probabilistic sensitivity analyses and the implications on the cost-effectiveness analysis^4–6^. However, modeling uncertainty is often overlooked, although it may have a much greater impact on results than parameter or methodological uncertainty^3^. Calibration methods address this issue connecting parameter inputs, structure, and outputs to identify the best fitting set of inputs within the margin of the uncertainty, or multiple sets of values, that mimic some specific empirical data. These values are then used to generate health and economic outputs for different interventions that are the basis for performing cost-effectiveness analysis and to provide decision-makers with policy-relevant data on the choices to be made. Therefore, appropriate input parameter estimates are critical if the model is to produce reliable and accurate results. Input parameters are generally informed by data available from published literature, clinical trials and expert opinion. However, results may vary across data sources and, on occasion, the data sources may not provide all the required inputs or are inaccurate, and sometimes natural history is not delineated in sufficient detail. Therefore, calibration of model inputs to known epidemiological endpoints informed by existing data can be a useful tool to ensure credibility of the results. Commonly used steps in the calibration process include identifying calibration targets, selecting measures of goodness-of-fit (GoF), defining the parameter space, selecting a search strategy, defining convergence thresholds, and specifying a stopping rule. Usual calibration targets include overall and disease-specific mortality and incidence rates.

In the context of cervical cancer, given the multiple prevention strategies available, mathematical models are a common tool to address different policy questions depending on the setting. Human papillomavirus (HPV), a common sexually transmitted infection, is considered as a necessary cause of cervical cancer. In fact, most men and women are infected with HPV at some time in their lives. Although most HPV infections resolve spontaneously, some can lead to formation of cervical abnormalities called cervical intraepithelial neoplasia (CIN), which can lead to cervical cancer, the second most common cancer in women worldwide^7–11^. These CIN, or precursor cervical lesions, can be further categorized depending on the degree of severity as CIN1 (mild abnormality), CIN2 (moderate to marked abnormality) or CIN3 (severe abnormality). Cervical cancer is a highly preventable disease by means of screening to find any precancerous lesions so they can be treated or by HPV vaccination to prevent infection from some of the most frequent high-risk types. At this time, the optimum prevention strategy is probably a combination of both interventions where specific parameters depend on the individual scenario. In Spain, cervical cancer prevention is based on opportunistic screening mainly based on cytology and the incorporation over the past years of an HPV vaccination program in preadolescent girls.

We used a Markov model that simulates the natural history of human papillomavirus (HPV) infection and subsequent cervical disease to evaluate different prevention strategies in Spain. The model generates health and economic outcomes such as cases averted, life expectancy (LE) from 11 years, reduction in the lifetime risk of CC, life years saved, quality-adjusted life years (QALYs), net health benefits, and lifetime costs for each prevention strategy. A robust calibration methodology is needed that can address the simultaneous estimations of these outcomes. The objective of this study is to explore the impact of some calibration approaches with different accuracy of initial inputs in terms of relative deviation from recorded Spanish epidemiological targets, in terms of health and economic outcomes, and also in the interpretation of cost-effectiveness analysis.

## Methods

### Model structure

Details of the Markov model structure have been previously described^12^. In short, a discrete-time, stochastic Markov chain model that simulates the natural history of HPV infection and cervical cancer was constructed. The basic model consists of 12 mutually exclusive and collectively exhaustive health states (a diagram is available as supplementary material) [healthy, HPV infection, CIN1-3 lesions, International Federation of Gynecology and Obstetrics (FIGO) cervical cancer stages, cancer survival, cervical cancer death, and death from other causes]. Death states (both from cervical cancer and other causes) reflect country-specific female mortality stratified by age. This closed model follows a single cohort of 11-year-old girls until they reach the age of 85 years or death using equal 1-year increments, where every woman has her own probability of progressing, regressing, or remaining at the same health state. All women start model simulations as healthy and can move to the HPV-infected state by acquiring the infection with certain probability. If a woman shows clearance of the infection, she will regress to the healthy state and then, reinfection is possible. If the infection persists, the woman will move into the CIN1 state and may then progress to CIN2 and later to CIN3 and cancer, or can regress and show clearance of the infection. Once in the cancer state, a woman may not regress to other health states, and instead progresses through the four stages of cancer according to the FIGO classification. A woman may die from cervical cancer if she belongs to the cancer stages or may die at any time from other noncervical cancer cause. Nonetheless, every woman has a certain probability of developing symptoms and receiving treatment. After treatment, a woman can return to the healthy state –if she belonged to one of the CIN2-3 states– or go to the cancer survival state –if she belonged to one of the FIGO states. Yearly regression and progression transition probabilities between health states were extracted from a literature review^13–17^. For each scenario and calibration method, 100 cohorts consisting of 100,000 women were simulated and a pooled cohort was analysed.

### Calibration

The calibration process is used to compare the values obtained from the model with those known empirical data to assess accuracy. For that purpose, it is usually calculated a statistical measure of goodness of fit that summarizes the discrepancy between observed and predicted values. One of the most frequently goodness-of-fit measures is the mean absolute percentage deviation (MAPD) of model-predicted endpoints from target estimates, including age-specific high-risk HPV prevalence and age-specific cervical cancer incidence:

1 $$\sum _{i\mathrm{=1}}^{2}\frac{|es{t}_{i}-ob{s}_{i}|}{ob{s}_{i}},$$

where *est*_*i*_ are the model based estimates of the *i*-th endpoint and *obs*_*i*_ are the data based target values of the *i*-th endpoint. If the importance of the considered outcomes is not uniform, a weighted version of (1) could be used as well, as in^18^. Computing time was also measured for each calibration approach.

The considered calibration methods are among the most used^18^, including Nelder-Mead algorithm, controlled random search (CRS) algorithm, manual calibration and no calibration. A manually calibrated matrix for Spanish data was available as it was previously obtained and used to produce the results reported in^12^. Two non-calibrated matrices were built by randomly modifying the manually calibrated matrix. The impossible transitions and absorbing states remained impossible and absorbing in the non-calibrated matrices, while all other probabilities were randomly generated to be at most 20% or 80% away from the corresponding probability in the manually calibrated matrix.

The Nelder-Mead algorithm is a direct-search algorithm based on geometric transformations of a non-degenerative simplex –an evolving set of parameter sets that seeks to move toward a better fit with each iteration. Advantages of the Nelder-Mead approach include its efficiency when dealing with a large number of parameters –which is key in our context–, as it is an heuristic method that works on a geometrical basis and there is no need to calculate derivatives of the objective function, so it is relatively easy to implement and use^19^. The controlled random search algorithms are similar to genetic algorithms^20^, as both start with a random population of points, and randomly evolve these points by heuristic rules. The procedure used in this work is direct (it does not involve gradients) and is applicable to constrained optimization^21^.

All the calibration methods were build using R^22^, and the developed scripts are available as supplementary material. For controlled random search, package nloptr was used^23^ while base function optim was used for Nelder-Mead search algorithm based calibration. In general, we considered the problem of finding the global minimum of the function (1).

### Prevention strategies

We considered different scenarios of vaccination alone, screening alone and combined vaccination and screening depending on the programme type, the frequency and, the age of starting and ending screening. Simulated strategies reflect in the most reliable possible way the current situation in Spain and the potential introduction of HPV primary screening. Intervention characteristics are defined hereafter.

#### No intervention

A reference scenario without vaccination or screening.

#### Vaccination alone

We assume that preadolescent girls are successfully vaccinated at the age of 12 years with three doses of the vaccine against HPV types 16 and 18. The analysis was carried out assuming favorable vaccine with 100% efficacy and lifelong duration of vaccine immunity to prevent cervical lesions caused by HPV 16 and 18 among uninfected women. No cross-protection against other high-risk HPV types was assumed. The uptake is set to 70%.

#### Screening alone

Screening scenarios may differ by screening test (cytology or HPV DNA testing), assuming a frequency of 3 years for cytology (3y-cytology) and 5 years for HPV (5y-HPV), targeted ages (25 to 65 years old), and switch age from cytology to HPV testing at 35 years old. Screening coverage is set to 70%. On the basis of a study carried out in Spain^24^ and the most up-to-date available information, we assume that the sensitivity and specificity of cytology to detect CIN2 is 38.2 and 97.4% respectively. Regarding CIN3, we set the sensitivity and specificity of cytology to 52.3 and 97.6%. Primary HPV DNA testing is performed in women older than 35 years of age with cytology triage for positive women. For women younger than 35 years of age, cytology is the reference test. The sensitivity and specificity of HPV DNA testing to detect CIN2 is 82.4 and 92.4%, respectively; 98 and 92.3% to detect CIN3 and 90.5 and 91.9% to detect CIN2+ for cytology after a positive HPV test^24^. Two screening scenarios have been defined, an organized framework assuming that all women are screened within the indicated period and opportunistic, where all parameters are set as before but women are supposed to be screened with different frequency. In the setting for this study, we assume that 15% of women are screened annually, 15% are screened each 2 years, 50% are screened each 3 years, 15% are screened each 4 years and 5% are screened each 5 years.

#### Combined vaccination and screening

In this scenario, we implement vaccination in girls aged 12 years, followed by screening according to the parameters and assumptions described previously for vaccination and screening alone. Both organized and opportunistic screening are conducted independently of vaccination status.

### Model outcomes

Given an input transition probabilities matrix, the model returns health and economic outputs such as the number of clinical procedures (HPV tests and cytologies), HPV prevalence, the number and incidence of CIN lesions according to severity, cancer cases and deaths, life expectancy, QALYs and total lifetime and per person costs for each considered prevention strategy. In turn, costs are split out in direct medical costs (including cost of cytology/HPV collection kit, complementary procedures, follow-up, treatments, staff, disposable supplies, laboratory transport, equipment, other supplies, and facilities), and direct non-medical costs (including patient transport and cost of patient time). From the information provided by the model, relevant information for scenario comparison as the number of cervical cancer cases averted, cost-effectiveness ratios (CERs), and incremental cost-effectiveness ratios (ICERs) respect the no intervention and the previous best non-weakly dominated strategy were obtained. For the purpose of comparing the results, percent change of CERs with respect to manual calibration and the average percent change and standard deviation of CERs were calculated.

The unitary cost per woman of the preventive interventions considered and treatment of premalignant lesions and cancer indexed at year 2016 is available as supplementary material. Indirect costs were not taken into account.

To be conservative, the willingness-to-pay threshold is defined at 20,000 €/QALY on the basis of the lowest values reported on the latest Spanish and European literature^25–27^.

All costs and health outcomes reported were discounted at an annual rate of 3%.

### Data availability statement

All data generated or analysed during this study are included in this published article (and its Supplementary Information files).

## Results

### Goodness of fit

Results show that when the original input matrix is close to the targeted data, no differences were observed between Nelder-Mead or controlled random search, therefore not much improvement is obtained after using an optimization algorithm. When the input matrix was far from fitting the observed data and no calibration process was carried out, the deviation from the target estimates was over 79% for the worst matrix and about 20% for the best. For the manually calibrated matrix the deviation was less than 2%, although it required more than 40 days of analyst work. Regarding automatically calibrated matrices, the deviation was about 7% and 5% with computation times of 7.3 hours and 52.3 hours for Nelder-Mead and controlled random search algorithms respectively starting with the good matrix, and similar deviation with computation times of 24.9 hours and over 100 hours starting with the worst matrix. These results are summarized in Table 1.

**Table 1:** Mean absolute percentage deviation (MAPD) and computing time in hours by calibration approach and input matrix (good and bad input matrices have a deviation about 20% and 80% from targeted values).

Calibration (matrix)	MAPD	Computing time (h)
No calibration (bad)	79.0%	—
No calibration (good)	20.0%	—
Manual	2.0%	1,008.0
Nelder-Mead (bad)	7.0%	24.9
Nelder-Mead (good)	6.9%	7.3
CRS (bad)	5.1%	102.0
CRS (good)	4.9%	52.3

**Figure 1:**
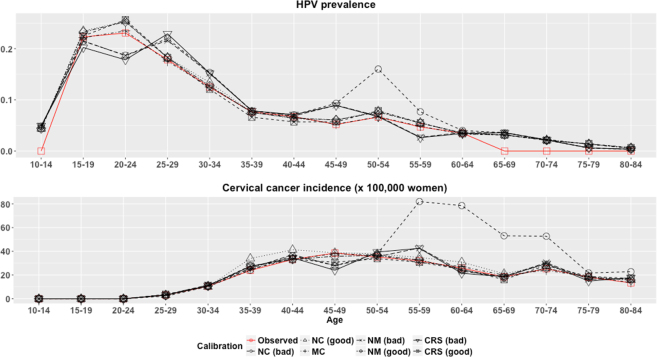
Model predicted values for age-specific HPV prevalence and cervical cancer incidence by calibration approach and input matrix. NC = No calibration, MC = Manual calibration, NM = Nelder-Mead, CRS = Controlled random search.

The observed HPV prevalence and CC incidence and the estimated by the model using each of the matrices are shown in Fig. 1, showing huge disturbances from targeted values when the input matrix was not calibrated. The manually calibrated matrix provides the best fitting to targeted values while non calibrated matrices produced the worst results.

### Cost-effectiveness outcomes

Regarding the main cost-effectiveness outcomes, the differences in discounted QALYs, discounted costs per person and CERs among the calibration methods can be seen in Table 2. The complete table including undiscounted values is provided as supplementary material.

**Table 2:** Cost-effectiveness outcomes by calibration approach and input matrix. LE = Life expectancy, NC = No calibration, MC = Manual calibration, NM = Nelder-Mead, CRS = Controlled random search.

Calibration	Strategy	QALYs	LE	% of cases averted	Cost (€)	CER (€/QALY)
NC (bad)	No intervention	29.7356	54.8909	—	107	3.6
	Vaccination	29.7418	54.9047	15.1	178	6.0
	5y-HPV (org)	29.7597	54.9419	26.0	268	9.0
	3y-cytology (org)	29.7593	54.9488	28.1	319	10.7
	3y-cytology (op)	29.7574	54.9360	21.4	354	11.9
	Vacc. + 5y-HPV (org)	29.7636	54.9491	37.6	326	11.0
	Vacc. + 3y-cytology (org)	29.7623	54.9542	39.0	393	13.2
	Vacc. + 3y-cytology (op)	29.7604	54.9434	34.0	425	14.3
NC (good)	No intervention	29.8237	54.8996	—	90	3.0
	Vaccination	29.8282	54.9129	14.7	162	5.4
	5y-HPV (org)	29.8480	54.9501	30.6	249	8.3
	3y-cytology (org)	29.8482	54.9544	29.2	309	10.4
	3y-cytology (op)	29.8472	54.9468	27.2	337	11.3
	Vacc. + 5y-HPV (org)	29.8492	54.9559	41.9	311	10.4
	Vacc. + 3y-cytology (org)	29.8495	54.9593	40.8	384	12.9
	Vacc. + 3y-cytology (op)	29.8486	54.9521	38.8	413	13.8
MC	No intervention	29.8631	54.9107	—	79	2.7
	Vaccination	29.9000	54.9438	44.5	130	4.4
	5y-HPV (org)	29.8856	54.9556	31.6	241	8.1
	3y-cytology (org)	29.8863	54.9587	30.2	301	10.1
	3y-cytology (op)	29.8836	54.9512	27.5	330	11.0
	Vacc. + 5y-HPV (org)	29.9128	54.9696	62.4	284	9.5
	Vacc. + 3y-cytology (org)	29.9128	54.9714	61.5	361	12.1
	Vacc. + 3y-cytology (op)	29.9128	54.9659	60.3	388	13.0
NM (bad)	No intervention	28.5862	54.9172	—	73	2.6
	Vaccination	28.7004	54.9265	14.6	149	5.2
	5y-HPV (org)	28.6086	54.9579	28.2	234	8.2
	3y-cytology (org)	28.6057	54.9609	27.9	283	9.9
	3y-cytology (op)	28.6076	54.9538	24.8	312	10.9
	Vacc. + 5y-HPV (org)	28.7166	54.9619	38.9	298	10.4
	Vacc. + 3y-cytology (org)	28.7162	54.9641	39.1	363	12.6
	Vacc. + 3y-cytology (op)	28.7142	54.9584	36.7	390	13.6
NM (good)	No intervention	29.7332	54.9085	—	78	2.6
	Vaccination	29.7439	54.9180	14.6	153	5.2
	5y-HPV (org)	29.7604	54.9533	30.1	241	8.1
	3y-cytology (org)	29.7601	54.9564	28.3	301	10.1
	3y-cytology (op)	29.7593	54.9499	26.5	329	11.1
	Vacc. + 5y-HPV (org)	29.7685	54.9585	41.1	304	10.2
	Vacc. + 3y-cytology (org)	29.7686	54.9615	39.8	377	12.7
	Vacc. + 3y-cytology (op)	29.7668	54.9552	38.4	406	13.6
CRS (bad)	No intervention	28.6707	54.9183	—	73	2.5
	Vaccination	28.7727	54.9279	14.3	149	5.2
	5y-HPV (org)	28.6925	54.9582	27.8	233	8.1
	3y-cytology (org)	28.6887	54.9609	27.9	283	9.9
	3y-cytology (op)	28.6889	54.9551	24.9	311	10.8
	Vacc. + 5y-HPV (org)	28.7885	54.9622	38.9	297	10.3
	Vacc. + 3y-cytology (org)	28.7900	54.9648	38.5	362	12.6
	Vacc. + 3y-cytology (op)	28.7902	54.9595	36.3	390	13.5
CRS (good)	No intervention	29.7306	54.9109	—	76	2.6
	Vaccination	29.7415	54.9209	15.2	151	5.1
	5y-HPV (org)	29.7568	54.9552	29.8	238	8.0
	3y-cytology (org)	29.7557	54.9575	27.7	299	10.0
	3y-cytology (op)	29.7551	54.9513	26.2	326	11.0
	Vacc. + 5y-HPV (org)	29.7648	54.9594	40.8	301	10.1
	Vacc. + 3y-cytology (org)	29.7642	54.9621	39.2	376	12.6
	Vacc. + 3y-cytology (op)	29.7636	54.9560	37.6	404	13.6

Table 2 shows that the largest differences in most cost-effectiveness outcomes correspond to the non-calibrated matrices as well. For instance, taking the manually calibrated matrix outcomes as a reference, the no intervention strategy CER for the non calibrated approach differs about 26 to 33% (for the good and bad input matrices respectively), being this difference about 4-5% for Nelder-Mead and CRS algorithms respectively.

**Figure 2:**
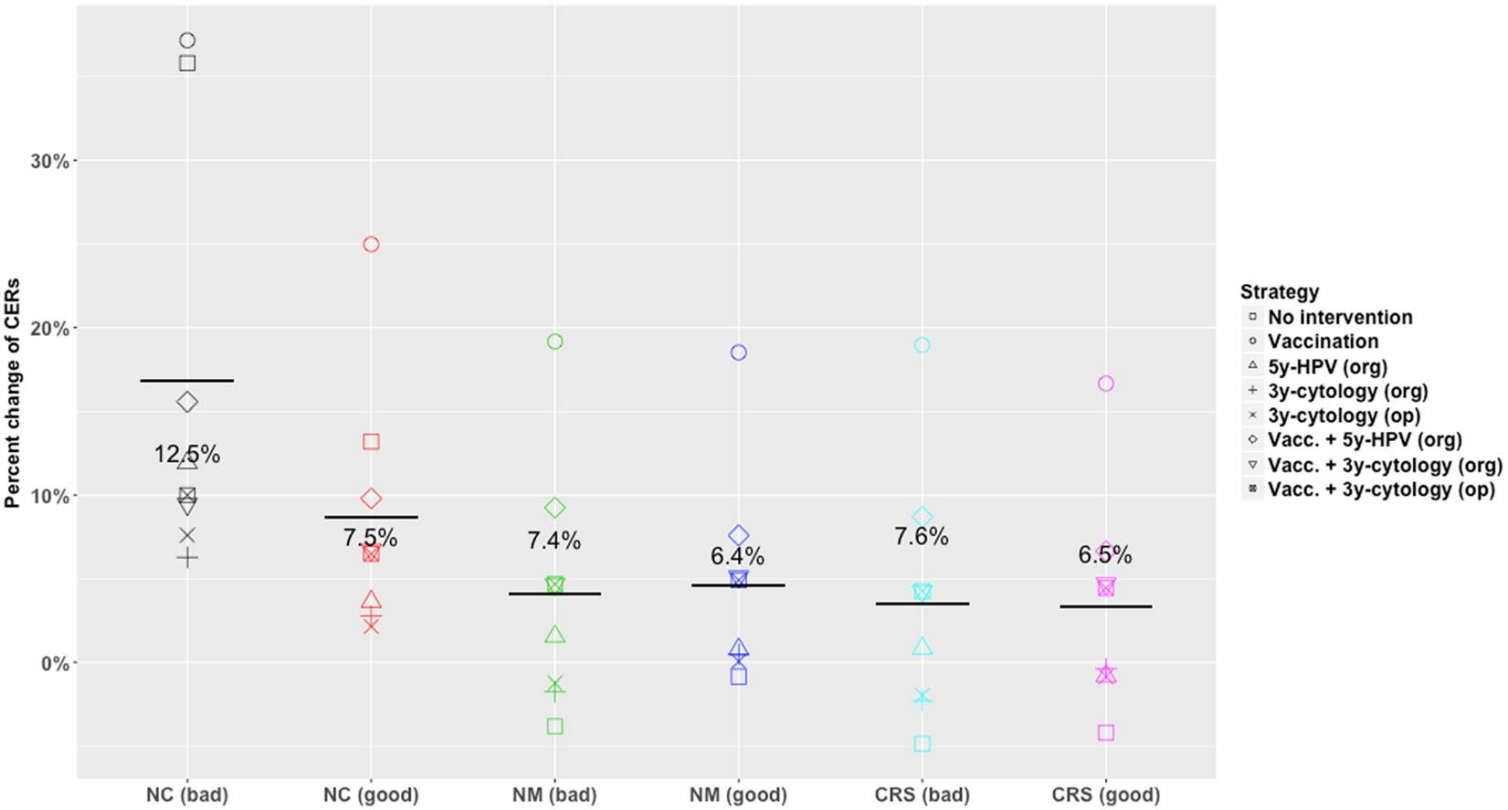
Percent change of CERs respect to manual calibration for each prevention strategy by calibration method and input matrix. The average percent change of CERs corresponds to the solid line and the standard deviation to the overprinted number. NC = No calibration, MC = Manual calibration, NM = Nelder-Mead, CRS = Controlled random search.

Figure 2 shows the percentage change of CERs for each strategy depending on the calibration method and input matrix with respect to the manually calibrated matrix and the overall average (solid black line) and standard deviation (overprinted number) by calibration method. It can be seen that the results that are more similar to those obtained using the manually calibrated matrix correspond to Nelder-Mead and CRS calibrated matrices, when the input matrix was relatively well calibrated. Taking all prevention strategies into account, the average relative change of CER from the manually calibrated matrix outcomes is 16.8%, 8.7%, 4.1%, 4.6%, 3.5% and 3.3% for non-calibrated (bad input matrix), non-calibrated (good input matrix), Nelder-Mead (bad input matrix), Nelder-Mead (good input matrix), CRS (bad input matrix) and CRS (good input matrix) respectively.

**Figure 3:**
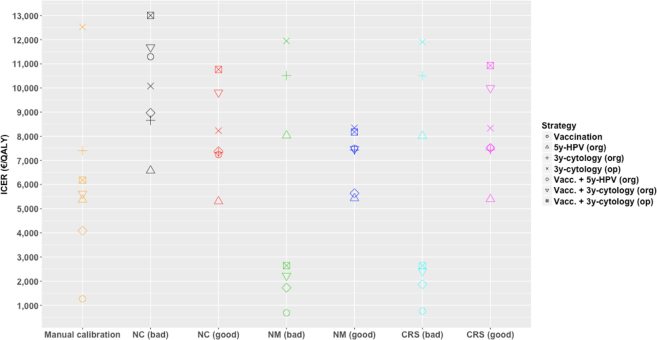
Incremental cost-effectiveness ratios with respect to no intervention scenario by calibration approach and input matrix. NC = No calibration, MC = Manual calibration, NM = Nelder-Mead, CRS = Controlled random search.

Figure 3 shows the ICERs calculated with respect to the no intervention scenario. It can be seen that ICERs present an erratic behavior and that they do not present a similar pattern, either with the order of the strategies or in terms of magnitude of the ratios, even the dispersion of the ICERs is different between calibration methods.

The incremental cost-effectiveness analysis comparing the considered prevention strategies depending on the calibration approach and input matrix is summarized in Table 3. As with the ICERs respect to no intervention, no clear pattern can be detected among the different calibration approaches although the cost-effectiveness analysis is quite uniform in its direction, despite the differences in ICERs magnitude. Three of the considered calibration approaches (manual, Nelder-Mead and CRS with the worst input matrix) point to cost-effectiveness of vaccination strategy, although the manual calibration double the costs compared to the other approaches. For all calibration methods, both opportunistic and organized cytology are strongly dominated (more costly and less effective than other strategies) or weakly dominated (with higher ICERs than more effective strategies), and the most cost-effective strategy is the combination of vaccination and organized HPV test screening every 5 years except for no calibration method using the best input matrix. Regarding this strategy, big differences in ICERs magnitude compared with the previous best non-dominated strategy can be seen among the considered calibration methods, ranging from 7,655 €/QALY to 52,692 €/QALY and being the results obtained through non calibrated matrices the ones resulting in greater ICERs. Nelder-Mead and CRS approaches lead to similar ICERs. The combination of vaccination and cytology strategies are over the willingness-to-pay threshold in all cases.

**Table 3:** Incremental cost-effectiveness ratios by calibration approach and input matrix. NC = No calibration, MC = Manual calibration, NM = Nelder-Mead, CRS = Controlled random search. dom = weakly dominated strategy.

Strategy	Incremental cost-effectiveness ratios (€/QALY)						
	NC (bad)	NC (good)	MC	NM (bad)	NM (good)	CRS (bad)	CRS (good)
No intervention							
Vaccination	dom	dom	1,372	662	dom	749	dom
5y-HPV (org)	6,665	6,560	dom	dom	5,990	dom	6,155
3y-cytology (org)	dom	dom	dom	dom	dom	dom	dom
3y-cytology (op)	dom	dom	dom	dom	dom	dom	dom
Vacc. + 5y-HPV (org)	14,746	52,692	12,061	9,206	7,655	9,390	7,964
Vacc. + 3y-cytology (org)	dom	211,657	>4M	dom	>1 M	41,922	dom
Vacc. + 3y-cytology (op)	dom	dom	>4 M	dom	dom	181,784	dom

## Discussion

Our analyses suggest that important differences in both goodness of fit and cost-effectiveness outcomes are found depending on the calibration approach and input matrix. The non calibrated matrices produced HPV prevalence and CC incidence curves absolutely far away from the target values, and the better results in most cost-effectiveness outcomes, most similar to those obtained by means of the manually calibrated matrix, correspond to the Nelder-Mead and CRS calibrated matrices using reasonable starting points. There is no clear pattern for ICERs across the different calibration approaches, probably due to the fact that there is no consistency in the direction and proportion of the difference among QALYs and costs. For instance, with bad input matrices, Nelder-Mead and CRS show a large decrease in the QALYs while costs remain similar, leading to important differences in ICERs. In our case, all calibration methods except the results coming from non calibrated matrices, point to the same strategy as the most cost-effective (combination of vaccination and organized screening with HPV testing every 5 years), but ICERs values are widely scattered. These variations in certain circumstances could involve different policy decisions determined by the ICER threshold selected. Therefore, the importance of a reliable calibration process is critical to get trustable cost-effectiveness results that finally provide the decision makers with the most accurate information.

Although the best fitting to targeted values was obtained by manual calibration, this approach requires a huge amount of time to produce a reasonable approximation. The advantage of this approach is that one could stop the calibration process when the desired deviance is obtained. However, very reasonable results can be obtained by automated optimization algorithms as Nelder-Mead, controlled random search or genetic algorithms. In our setting, Nelder-Mead algorithm obtained very reasonable results in a relatively short time, especially if the original matrix is not dramatically far from the targeted values. By means of CRS algorithm a slightly lower deviation was achieved but much more time was needed and the results are very similar to Nelder-Mead algorithm calibrated matrices, which seems to be the best alternative in terms of efficiency.

Markov models are useful for modeling complex health interventions and support problems involving policy decisions. However, it suffers from the usual limitations of Markov models^28^. The main limitation of the present study might be that it is based only in one matrix for each kind of calibration approach, due to the large computing time of the automated algorithms. Although no significant differences with the present work might be expected, it would be interesting to explore thoroughly the performance of the automated methods, specially Nelder-Mead algorithm, by means of a set of randomly generated matrices.

The results obtained in our study are similar to those reported in Taylor *et al*.^18^, in the sense that a calibration based on the Nelder-Mead algorithm or a strategy combining manual calibration and Nelder-Mead algorithm arises as the best approaches, being capable of providing accurate results in a reasonable amount of time. However, Taylor *et al*.^18^ focuses on the goodness of fit of the calibration approaches while we highlight the cost-effectiveness analysis point of view because the final goal of the calibration process is to use the best possible input for the health decision-making.

This work shows that a thorough verification of the calibration process is essential in a decision-making framework, as large differences can be obtained on health benefits and costs among calibration approaches, with an unknown impact on cost-effectiveness analyses that might lead to non-optimal decisions.

## Electronic supplementary material

Supplementary material

## References

[1] Drummond, M., Stoddart, G. & Torrance, G. *Methods for the economic evaluation of health care programmes* (Oxford Medical Publications, 1987), first edn.

[2] Kim, S. Y., Russell, L. B. & Sinha, A. Handling parameter uncertainty in cost-effectiveness models simply and responsibly. *Medical Decision Making* 567–569, 10.1177/0272989X14567475. 10.1177/0272989X14567475 26280060

[3] Brisson, M. & Edmunds, W. J. Impact of model, methodological, and parameter uncertainty in the economic analysis of vaccination programs. *Medical Decision Making* 434–446, 10.1177/0272989X06290485. 10.1177/0272989X06290485 16997923

[4] Stout, N. K., Knudsen, A. B., Kong, C. Y., McMahon, P. M. & Gazelle, G. S. Calibration methods used in cancer simulation models and suggested reporting guidelines. *PharmacoEconomics* **27**, 533–45, http://link.springer.com/10.2165/11314830-000000000-00000, 10.2165/11314830-000000000-00000 (2009). 10.2165/11314830-000000000-00000 PMC2787446 19663525

[5] Kim, J. J. *et al*. Multiparameter calibration of a natural history model of cervical cancer. *American Journal of Epidemiology* **166**, 137–150, https://academic.oup.com/aje/article-lookup/doi/10.1093/aje/kwm086, 10.1093/aje/kwm086 (2007). 10.1093/aje/kwm086 17526866

[6] Van de Velde, N., Brisson, M. & Boily, M. C. Modeling human papillomavirus vaccine effectiveness: quantifying the impact of parameter uncertainty. *American Journal of Epidemiology* **165**, 762–75, https://academic.oup.com/aje/article-lookup/doi/10.1093/aje/kwk059, 10.1093/aje/kwk059 (2007). 10.1093/aje/kwk059 17276976

[7] Muñoz N (1992). The causal link between human papillomavirus and invasive cervical cancer: a population-based case-control study in Colombia and Spain. International journal of cancer.

[8] Bosch, F. X., Lorincz, A., Muñoz, N., Meijer, C. J. L. M. & Shah, K. V. The causal relation between human papillomavirus and cervical cancer. *Journal of Clinical Pathology* **55**, 244–265, http://jcp.bmj.com/content/55/4/244 (2002). 10.1136/jcp.55.4.244 PMC1769629 11919208

[9] Schiffman MH (1993). Epidemiologic evidence showing that human papillomavirus infection causes most cervical intraepithelial neoplasia. Journal of the National Cancer Institute.

[10] Kjær SK (1996). Human papillomavirus—the most significant risk determinant of cervical intraepithelial neoplasia. International Journal of Cancer.

[11] Parkin, D. M., Bray, F., Ferlay, J. & Pisani, P. Estimating the world cancer burden: Globocan 2000. *International journal of cancer* **94**, 153–6, http://www.ncbi.nlm.nih.gov/pubmed/11668491 (2001). 10.1002/ijc.1440 11668491

[12] Georgalis L, De Sanjosé S, Esnaola M, Bosch FX, Diaz M (2015). Present and future of cervical cancer prevention in Spain: A cost-effectiveness analysis. European Journal of Cancer Prevention.

[13] Myers ER, McCrory DC, Nanda K, Bastian L, Matchar DB (2000). Mathematical model for the natural history of human papillomavirus infection and cervical carcinogenesis. American journal of epidemiology.

[14] Canfell, K., Barnabas, R., Patnick, J. & Beral, V. The predicted effect of changes in cervical screening practice in the UK: Results from a modelling study. *British journal of cancer* **91**, 530–6, http://www.pubmedcentral.nih.gov/articlerender.fcgi?artid=PMC2409838, 10.1038/sj.bjc.6602002 (2004). 10.1038/sj.bjc.6602002 PMC2409838 15266332

[15] Insinga, R. P., Glass, A. G., Myers, E. R. & Rush, B. B. Abnormal outcomes following cervical cancer screening: Event duration and health utility loss. *Medical Decision Making* **27**, 414–422, http://mdm.sagepub.com/cgi/doi/10.1177/0272989X07302128, 10.1177/0272989X07302128 (2007). 10.1177/0272989X07302128 17585005

[16] Kulasingam, S. L., Benard, S., Barnabas, R. V., Largeron, N. & Myers, E. R. Adding a quadrivalent human papillomavirus vaccine to the UK cervical cancer screening programme: A cost-effectiveness analysis. *Cost effectiveness and resource allocation* **6**, 4, https://www.ncbi.nlm.nih.gov/pmc/articles/PMC2290741/, 10.1186/1478-7547-6-4 (2008). 10.1186/1478-7547-6-4 PMC2290741 18279515

[17] Kohli, M., Lawrence, D., Haig, J., Anonychuk, A. & Demarteau, N. Modeling the impact of the difference in cross-protection data between a human papillomavirus (HPV)-16/18 AS04-adjuvanted vaccine and a human papillomavirus (HPV)-6/11/16/18 vaccine in Canada. *BMC public health* **12**, 872, http://www.biomedcentral.com/1471-2458/12/872, 10.1186/1471-2458-12-872 (2012). 10.1186/1471-2458-12-872 PMC3503751 23061913

[18] Taylor DCA (2010). Methods of model calibration. PharmacoEconomics.

[19] Lewis, R. M., Torczon, V. & Trosset, M. W. Direct search methods: then and now. *Journal of Computational and Applied Mathematics* **124**, 191–207 http://www.sciencedirect.com/science/article/pii/S0377042700004234, 10.1016/S0377-0427(00)00423-4 (2000).

[20] Sivanandam, S. N. & Deepa, S. N. *Introduction to Genetic Algorithms*, 1st edn (Springer Publishing Company, Incorporated, 2007).

[21] Price WL (1983). Global optimization by controlled random search. Journal of Optimization Theory and Applications.

[22] R CoreTeam. R: *A language and environment for statistical computing* https://www.r-project.org/ (2016).

[23] Johnson, S. G. *The nlopt nonlinear-optimization package*. http://ab-initio.mit.edu/nlopt.

[24] Ibáñez, R. *et al*. Protecting the underscreened women in developed countries: the value of HPV test. *BMC Cancer* **14**, 574 https://www.ncbi.nlm.nih.gov/pmc/articles/PMC4137095/, 10.1186/1471-2407-14-574 (2014). 10.1186/1471-2407-14-574 PMC4137095 25102758

[25] Vallejo-Torres, L., García-Lorenzo, B. & Serrano-Aguilar, P. Estimating a cost-effectiveness threshold for the spanish NHS https://ideas.repec.org/p/fda/fdaeee/eee2016-22.html (2016). 10.1002/hec.3633 29282798

[26] Bogaards JA, Coupé VMH, Meijer CJLM, Berkhof J (2011). The clinical benefit and cost-effectiveness of human papillomavirus vaccination for adult women in the Netherlands. Vaccine.

[27] Coupé, V. M. H., Bogaards, J. A., Meijer, C. J. L. M. & Berkhof, J. Impact of vaccine protection against multiple HPV types on the cost-effectiveness of cervical screening. *Vaccine* **30**, 1813–1822, 10.1016/j.vaccine.2012.01.001 (2012). 10.1016/j.vaccine.2012.01.001 22240341

[28] Sonnenberg, F. A. & Beck, J. R. Markov models in medical decision making. *Medical Decision Making* **13**, 322–338, 10.1177/0272989X9301300409 PMID: 8246705 (1993). 10.1177/0272989X9301300409 8246705

