# Supplementary material for: Impact of model calibration on cost-effectiveness analysis of cervical cancer prevention
Source: Sci Rep. 2017 Dec 8;7:17208. doi: 10.1038/s41598-017-17215-2 (PMC5722890; doi:10.1038/s41598-017-17215-2)
Supplement: Supplementary file 1 — Supplementary material [file 41598_2017_17215_MOESM1_ESM.pdf]

SUPPLEMENTARY MATERIAL

Accompanying the manuscript:

Impact of model calibration on cost-effectiveness analysis of cervical cancer prevention

David Moriña[1*], Silvia de Sanjosé[2,3] and Mireia Diaz[1,4]

[1]Unit of Infections and Cancer (UNIC - I&I), Cancer Epidemiology Research Program (CERP), Catalan Institute of Oncology (ICO)-IDIBELL, L'Hospitalet de Llobregat, Barcelona, Spain

[2]Cancer Epidemiology Research Program (CERP), Catalan Institute of Oncology (ICO)-IDIBELL, L'Hospitalet de Llobregat, Barcelona, Spain

[3]Centro de Investigación Biomédica en Red (CIBERESP), Barcelona, Spain

[4]Centro de Investigación Biomédica en Red (CIBERONC), Barcelona, Spain

*dmorina@iconcologia.net

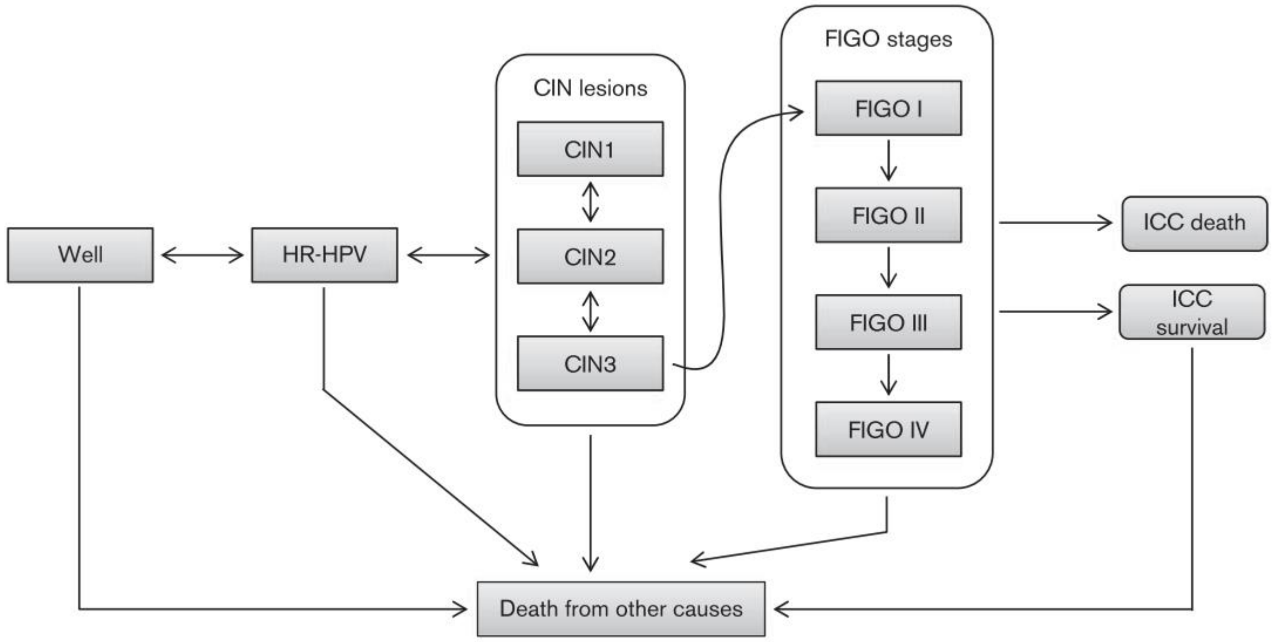

Figure S1. Diagram of the Markov model that reproduces the natural history of cervical cancer.

Age group	HPV prevalence
**10-14**	0.0000
**15-19**	0.2229
**20-24**	0.2307
**25-29**	0.1783
**30-34**	0.1250
**35-39**	0.0764
**40-44**	0.0671
**45-49**	0.0519
**50-54**	0.0663
**55-59**	0.0471
**60-64**	0.0348
**65-69**	0.0000
**70-74**	0.0000
**75-79**	0.0000
**80-84**	0.0000

Table S2. Observed age-specific high-risk HPV prevalence in Spain.

Age group	CC Incidence
**10-14**	0.000
**15-19**	0.000
**20-24**	0.000
**25-29**	3.128
**30-34**	10.400
**35-39**	23.876
**40-44**	33.084
**45-49**	38.568
**50-54**	35.160
**55-59**	31.980
**60-64**	25.824
**65-69**	18.396
**70-74**	25.304
**75-79**	18.178
**80-84**	13.344

Table S3. Observed age-specific cervical cancer incidence in Spain

Strategy	Description	Direct Medical cost	Direct non-medical cost
**Vaccination**	Cost of vaccination and administration fees per dose	34.3€	0.0€
**Cytology**	Cost of cytology collection kit, staff, disposable supplies, laboratory transport, equipment, other supplies, facilities, patient transport, and cost of patient time	56.8€	17.7€
**HPV test**	Cost of HPV collection kit, staff, disposable supplies, laboratory transport, equipment, other supplies, facilities, patient transport, and cost of patient time	63.9€	17.7€
**CIN 1**	This is a weighted average of the cost of follow-up and cytology if negative result or biopsy if positive result with cytology after 6 and 12 months or follow-up with colposcopy and cytology if negative result or biopsy if positive result	248.2€	79.5€
**CIN 2-3**	Cost of treating a person who has true CIN2-3. This includes the treatment-specific staff time, supplies, equipment, hospitalization and follow-up visits and procedures as well as patient time receiving services, hospitalization and follow-up and patient transport	1,461.0€	189.6€
**FIGO I**	Cost of staging and treatment for stage 1 cancer (local cancer). This includes patient time for follow-up visits, hospitalization, patient transport, complementary procedures (pap or colposcopy)	5,417.3€	214.0€
**FIGO II**	Cost of staging and treatment for stage 2 cancer (regional cancer). This includes patient time for follow-up visits, hospitalization, patient transport, complementary procedures (pap or colposcopy)	12,136.3€	214.0€
**FIGO III**	Cost of staging and treatment for stage 3 cancer (distant cancer). This includes patient time for follow-up visits, hospitalization, patient transport, complementary procedures (pap or colposcopy)	22,583.6€	214.0€
**FIGO IV**	Cost of staging and treatment for stage 4 cancer. This includes patient time for follow-up visits, hospitalization, patient transport, complementary procedures (pap or colposcopy)	33,222.5€	214.0€

Table S4. Unitary cost per woman of vaccination, screening tests, follow-up or treatment of premalignant lesions, and treatment of cervical cancer stages (costs indexed at year 2016).

Calibration	Scenario	Change of CER
**NC (bad)**	No intervention	0.35787
	Vaccination	0.37151
	3y-cytology (org)	0.06275
	3y-cytology (op)	0.07595
	5y-HPV (org)	0.11925
	Vacc. + 3y-cytology (org)	0.09450
	Vacc. + 3y-cytology (op)	0.09986
	Vacc. + 5y-HPV (org)	0.15553
**NC (good)**	No intervention	0.13195
	Vaccination	0.24971
	3y-cytology (org)	0.02763
	3y-cytology (op)	0.02145
	5y-HPV (org)	0.03620
	Vacc. + 3y-cytology (org)	0.06716
	Vacc. + 3y-cytology (op)	0.06484
	Vacc. + 5y-HPV (org)	0.09797
**NM (bad)**	No intervention	-0.03810
	Vaccination	0.19157
	3y-cytology (org)	-0.01755
	3y-cytology (op)	-0.01238
	5y-HPV (org)	0.01547
	Vacc. + 3y-cytology (org)	0.04660
	Vacc. + 3y-cytology (op)	0.04673
	Vacc. + 5y-HPV (org)	0.09241
**NM (good)**	No intervention	-0.00852
	Vaccination	0.18497
	3y-cytology (org)	0.00463
	3y-cytology (op)	0.00050
	5y-HPV (org)	0.00775
	Vacc. + 3y-cytology (org)	0.05113
	Vacc. + 3y-cytology (op)	0.04903
	Vacc. + 5y-HPV (org)	0.07582
	No intervention	-0.04852
**CRS (bad)**	Vaccination	0.18939
	3y-cytology (org)	-0.02278
	3y-cytology (op)	-0.01959
	5y-HPV (org)	0.00831
	Vacc. + 3y-cytology (org)	0.04236
	Vacc. + 3y-cytology (op)	0.04249
	Vacc. + 5y-HPV (org)	0.08690
**CRS (good)**	No intervention	-0.04188
	Vaccination	0.16643
	3y-cytology (org)	-0.00403
	3y-cytology (op)	-0.00847
	5y-HPV (org)	-0.00820
	Vacc. + 3y-cytology (org)	0.04694
	Vacc. + 3y-cytology (op)	0.04436
	Vacc. + 5y-HPV (org)	0.06638

Table S5. Percent change of CERs (discounted) respect to manual calibration for each prevention strategy by calibration method and input matrix. NC=No calibration, MC=Manual calibration, NM=Nelder-Mead, CRS=Controlled random search.

Calibration	Strategy	ICER (€/QALY)
**NC (bad)**	Vaccination	11,292.6
	3y-cytology (org)	8,657.4
	3y-cytology (op)	10,079.9
	5y-HPV (org)	6,576.9
	Vacc. + 3y-cytology (org)	11,684.7
	Vacc. + 3y-cytology (op)	13,006.4
	Vacc. + 5y-HPV (org)	8,967.0
**NC (good)**	Vaccination	7,243.4
	3y-cytology (org)	7,315.5
	3y-cytology (op)	8,230.4
	5y-HPV (org)	5,304.0
	Vacc. + 3y-cytology (org)	9,811.6
	Vacc. + 3y-cytology (op)	10,766.4
	Vacc. + 5y-HPV (org)	7,376.9
**Manual calibration**	Vaccination	1,267.0
	3y-cytology (org)	7,397.3
	3y-cytology (op)	12,527.0
	5y-HPV (org)	5,370.3
	Vacc. + 3y-cytology (org)	5,625.2
	Vacc. + 3y-cytology (op)	6,180.0
	Vacc. + 5y-HPV (org)	4,089.6
**NM (bad)**	Vaccination	687.9
	3y-cytology (org)	10,511.5
	3y-cytology (op)	11,944.0
	5y-HPV (org)	8,032.5
	Vacc. + 3y-cytology (org)	2,225.6
	Vacc. + 3y-cytology (op)	2,642.7
	Vacc. + 5y-HPV (org)	1,728.2
**NM (good)**	Vaccination	7,495.0
	3y-cytology (org)	7,434.0
	3y-cytology (op)	8,346.7
	5y-HPV (org)	5,431.3
	Vacc. + 3y-cytology (org)	7,473.8
	Vacc. + 3y-cytology (op)	8,179.0
	Vacc. + 5y-HPV (org)	5,640.0
**CRS (bad)**	Vaccination	763.5
	3y-cytology (org)	10,505.0
	3y-cytology (op)	11,903.0
	5y-HPV (org)	8,012.0
	Vacc. + 3y-cytology (org)	2,411.1
	Vacc. + 3y-cytology (op)	2,643.4
	Vacc. + 5y-HPV (org)	1,870.6
**CRS (good)**	Vaccination	7,519.0
	3y-cytology (org)	7,435.3
	3y-cytology (op)	8,336.3
	5y-HPV (org)	5,392.0
	Vacc. + 3y-cytology (org)	9,998.7
	Vacc. + 3y-cytology (op)	10,928.7
	Vacc. + 5y-HPV (org)	7,515.7

Table S6. Incremental cost-effectiveness ratios (discounted) with respect to no intervention scenario by calibration approach andinput matrix. NC=No calibration, MC=Manual calibration, NM=Nelder-Mead, CRS=Controlled random search.

Calibration	Scenario	QALYs	LE	% of cases	Cost	CER
**NC (bad)**	No intervention	70.0090	74.8671	-	470	6.7
	Vaccination	70.0192	74.8859	15.1	485	6.9
	5y-HPV (org)	70.1112	74.9367	26.0	814	11.6
	3y-cytology (org)	70.1129	74.9461	28.1	981	14.0
	3y-cytology (op)	70.0987	74.9286	21.4	1049	15.0
	Vacc. + 5y-HPV (org)	70.1110	74.9465	37.6	827	11.8
	Vacc. + 3y-cytology (org)	70.1089	74.9535	39.0	1016	14.5
	Vacc. + 3y-cytology (op)	70.0962	74.9388	34.0	1072	15.3
**NC (good)**	No intervention	70.5603	74.8790	-	336	4.8
	Vaccination	70.5657	74.8971	14.7	372	5.3
	5y-HPV (org)	70.6584	74.9479	30.5	697	9.9
	3y-cytology (org)	70.6596	74.9537	29.2	898	12.7
	3y-cytology (op)	70.6507	74.9434	27.2	928	13.1
	Vacc. + 5y-HPV (org)	70.6473	74.9558	41.9	731	10.3
	Vacc. + 3y-cytology (org)	70.6513	74.9605	40.8	944	13.4
	Vacc. + 3y-cytology (op)	70.6448	74.9506	38.8	976	13.8
**MC**	No intervention	70.7883	74.8941	-	301	4.3
	Vaccination	70.9020	74.9393	44.5	254	3.6
	5y-HPV (org)	70.8788	74.9553	31.6	669	9.4
	3y-cytology (org)	70.8829	74.9596	30.2	873	12.3
	3y-cytology (op)	70.8696	74.9494	27.4	903	12.7
	Vacc. + 5y-HPV (org)	70.9531	74.9744	62.4	636	9.0
	Vacc. + 3y-cytology (org)	70.9543	74.9769	61.5	863	12.2
	Vacc. + 3y-cytology (op)	70.9526	74.9695	60.3	889	12.5
**NM (bad)**	No intervention	66.4356	74.9030	-	281	4.2
	Vaccination	66.7680	74.9156	14.6	326	4.9
	5y-HPV (org)	66.5186	74.9585	28.2	646	9.7
	3y-cytology (org)	66.5147	74.9627	27.9	820	12.3
	3y-cytology (op)	66.5140	74.9530	24.8	858	12.9
	Vacc. + 5y-HPV (org)	66.8308	74.9640	38.9	686	10.3
	Vacc. + 3y-cytology (org)	66.8330	74.9670	39.1	880	13.2
	Vacc. + 3y-cytology (op)	66.8213	74.9592	36.7	912	13.6
**NM (good)**	No intervention	69.9911	74.8912	-	294	4.2
	Vaccination	70.0325	74.9042	14.6	337	4.8
	5y-HPV (org)	70.1090	74.9523	30.1	669	9.5
	3y-cytology (org)	70.1084	74.9565	28.3	869	12.4
	3y-cytology (op)	70.1009	74.9477	26.5	899	12.8
	Vacc. + 5y-HPV (org)	70.1397	74.9593	41.1	705	10.1
	Vacc. + 3y-cytology (org)	70.1385	74.9634	39.8	920	13.1
	Vacc. + 3y-cytology (op)	70.1302	74.9548	38.4	950	13.5
**CRS (bad)**	No intervention	66.5797	74.9050	-	278	4.2
	Vaccination	66.8737	74.9166	14.3	325	4.9
	5y-HPV (org)	66.6594	74.9589	27.8	643	9.6
	3y-cytology (org)	66.6545	74.9634	27.9	818	12.3
	3y-cytology (op)	66.6478	74.9543	24.9	853	12.8
	Vacc. + 5y-HPV (org)	66.9338	74.9643	38.8	684	10.2
	Vacc. + 3y-cytology (org)	66.9414	74.9676	38.5	878	13.1
	Vacc. + 3y-cytology (op)	66.9371	74.9602	36.3	911	13.6
**CRS (good)**	No intervention	69.9440	74.8945	-	283	4.0
	Vaccination	69.9851	74.9081	15.2	328	4.7
	5y-HPV (org)	70.0574	74.9548	29.8	658	9.4
	3y-cytology (org)	70.0524	74.9580	27.6	864	12.3
	3y-cytology (op)	70.0458	74.9495	26.2	890	12.7
	Vacc. + 5y-HPV (org)	70.0862	74.9606	40.8	697	9.9
	Vacc. + 3y-cytology (org)	70.0834	74.9642	39.2	916	13.1
	Vacc. + 3y-cytology (op)	70.0768	74.9559	37.6	945	13.5

Table S7. Undiscounted cost-effectiveness outcomes by calibration approach and input matrix. NC=No calibration, MC=Manual calibration, NM=Nelder-Mead, CRS=Controlled random search.

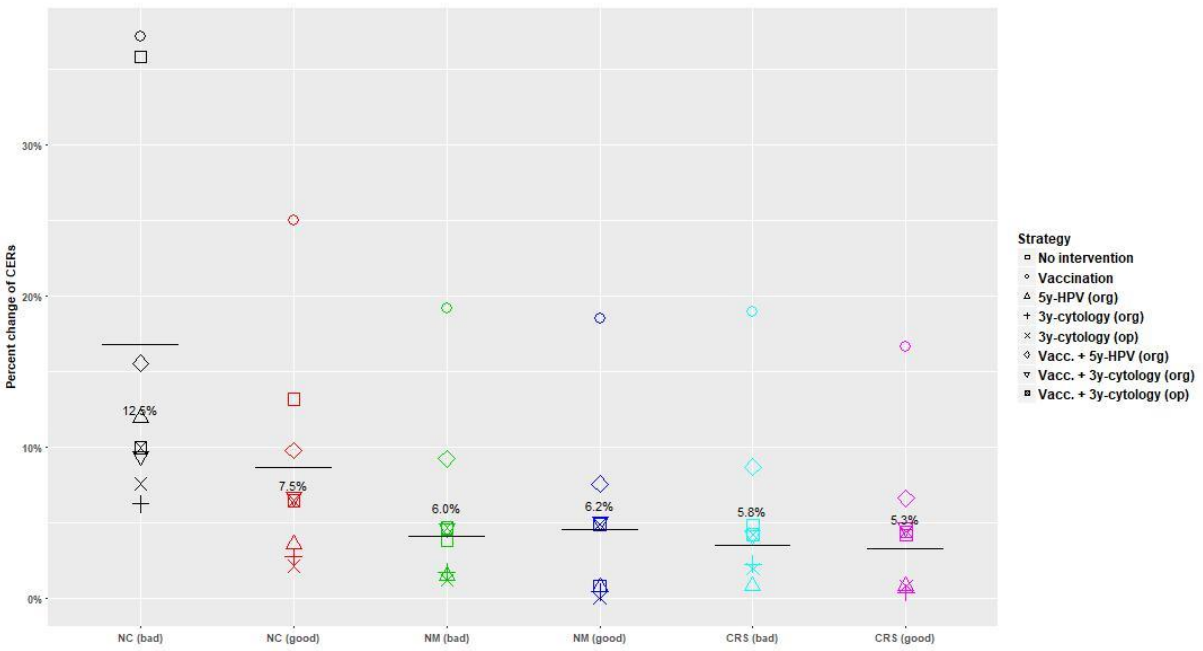

Figure S2. Absolute value of the percent change of CERs respect to manual calibration for each prevention strategy by calibration method and input matrix. The average percent change of CERs corresponds to the solid line and the standard deviation to the overprinted number. NC=No calibration, MC=Manual calibration, NM=Nelder-Mead, CRS=Controlled random search.

R code to reproduce the calibration process

```
NHvalidacrs <- function(probs, prev, inc, mort=NULL, N=500, keep=20, p.change, opt=FALSE)
{
 if (dev.cur() != 1) dev.off()
 p.change <- p.change/100
 if (N < keep) stop("Number of matrices to keep should be lower than the number of simulations")
 if (keep < 2) stop("The minimum is to perform 2 simulations")
 if (!all(prev[, 1] == inc[, 1])) stop("Age groups in reference prevalences/incidences should be equal")
 if (opt==TRUE)
 {
  res <- vector()
  pars <- as.vector(t(probs[, 2:dim(probs)[2]]))
  pars <- pars[pars != 0 & pars != 1]
  res2  <- crs2lm(pars, eucl.distance, rep(0.0000000001, length(pars)), rep(0.999999999999, length(pars)),
                  maxeval=1e5, prob=probs, prev=prev, inc=inc)
  print(res2$convergence)
  res <- res2$par
  probs.optim <- probs
  k <- 1
  for (i in 1:dim(probs)[1])
  {
   for (j in 2:dim(probs)[2])
   {
    if (probs[i, j] == 0 | probs[i, j] == 1)
    {
     probs.optim[i, j] <- probs[i, j]
    }else{
     probs.optim[i, j] <- res[k]
     k <- k + 1
    }
   }
  }
  probs      <- probs.optim
  probs2      <- cbind(probs[, 1], t(apply(probs[, 2:dim(probs)[2]], 1, function(x)(x/sum(x))))) ### Normalize each row probability to sum 1
  probs2      <- as.data.frame(probs2)
  probs2[, 1] <- probs[, 1]
```

```
  colnames(probs2)[1] <- colnames(probs)[1]
  probs      <- probs2
}
probs.def   <- vector("list", keep)
prevalences <- vector("list", keep)
incidences  <- vector("list", keep)
mortality   <- vector("list", keep)
dists       <- vector()
prevalence1 <- function(x){
  num <- x[2]
  den <- sum(x[1:10])
  return(num/den)
}
incidence1 <- function(i, x, newCasesMean1){
  num <- newCasesMean1[i]
  den <- sum(x[i, c(1:10)])
  num/den
}

n <- 1
while(n <= N)
{
  probs.prova <- matrix(nrow=nrow(probs), ncol=ncol(probs))
  probs.prova[, 1] <- probs[, 1]
  for (i in 1:nrow(probs))
  {
    for (j in 2:ncol(probs))
    {
      if (probs[i, j] != 0 & probs[i, j] != 1)
      {
        probs.prova[i, j] <- sample(rpert(5000, min=max(as.numeric(probs[i, j])*(1-p.change), 0.0000000001), mode=as.numeric(probs[i, j]),
                                  max=min(c(as.numeric(probs[i, j])*(1+p.change), 0.999999999))), 1)
      }else{
        probs.prova[i, j] <- probs[i, j]
      }
    }
  }

  ### Standardize all rows to sum 1
  probs.prova <- cbind(probs.prova[, 1], t(apply(probs.prova[, 2:dim(probs.prova)[2]], 1, function(x)(x/(sum(x))))))
  probs.prova <- as.data.frame(probs.prova)
  probs.prova[, 1] <- probs[, 1]
  cat("Step: ", n, "\n")
  res <- simCohort(probs1=probs.prova, Nsim = 5,
              figoSymProb = c(.11, .23, .66, .9), ### Probability to develop symptoms in FIGO states
              vaccinePrice.md = 0, vaccinePrice.nmd = 0, vaccinePrice.i = 0, screenPrice.md = 0, screenPrice.nmd = 0, screenPrice.i = 0,
              costCoefs.md = c(0, 0, 248.2, 1461.0, 1461.0, 5417.3, 12136.3, 22583.6, 33222.5, 0, 0, 0),
              costCoefs.nmd = c(0, 0, 79.5, 189.6, 189.6, 214.0, 214.0, 214.0, 214.0, 0, 0, 0),
              costCoefs.i = c(0, 0, 0, 0, 0, 0, 0, 0, 0, 0, 0, 0),
              vaccineCov = 0,
              utilityCoefs = c(1, 1, 0.987, 0.87, 0.87, 0.76, 0.67, 0.67, 0.67, 0.938, 0, 0),
              screenProbs = c(0, 0, 1, 1, 1, 0.9688, 0.9066, 0.7064, 0.3986, 0, 0, 0), ndoses = 0)

  tmp <- lapply(as.list(unique(res$age)), function(i, dat){
    aux <- res[res$age == i, ]
    apply(aux[, 1:(ncol(aux)-2)], 2, mean)
  }, dat = res)
  meanDat1 <- as.data.frame(t(as.matrix(as.data.frame(tmp))))
  rownames(meanDat1) <- NULL

  totPrev1 <- apply(meanDat1, 1, prevalence1)

  newCasesMean1 <- apply(attr(res, "newCases"), 2, mean)

  names(newCasesMean1) <- 1:length(newCasesMean1)
  totInc1 <- unlist(lapply(as.list(1:nrow(meanDat1)), incidence1,
                  x = meanDat1, newCasesMean1 = newCasesMean1))

  CCdeathsMean1 <- apply(attr(res, "CCdeaths"), 2, mean)
```

```
  age  <- as.numeric(substr(probs[1, 1], 1, 2)):as.numeric(substr(probs[dim(probs)[1], 1], 4, 5))
  ages <- as.numeric(substr(prev[1, 1], 1, 2)):as.numeric(substr(prev[dim(prev)[1], 1], 4, 5))
  HNObsPrev <- approx(prev[, 2], n=length(ages))$y

  tmp1 <- NULL
  tmp2 <- NULL
  ageGroupPrev1_pr <- rep(NA, length(unique(probs[, 1])))
  currentAgeGroup <- 1
  i <- 1
  ageGroups <- unique(probs[, 1])
  for(year in age){
    if(year > as.numeric(substr(ageGroups[currentAgeGroup], 4, 5))){
      ageGroupPrev1_pr[currentAgeGroup] <- sum(tmp1)/sum(tmp2)
      currentAgeGroup <- currentAgeGroup + 1
      tmp1 <- NULL
      tmp2 <- NULL
    }
    tmp1 <- c(tmp1, meanDat1[i, 2])
    tmp2 <- c(tmp2, sum(meanDat1[i, 1:(dim(meanDat1)[2]-2)]))
    i <- i + 1
  }
  ageGroupPrev1_pr[length(unique(probs[, 1]))] <- sum(tmp1)/sum(tmp2)
  ageGroupNames <- unique(probs[, 1])

  tmp <- NULL
  tmp2 <- NULL
  ageGroupInc1 <- rep(NA, length(unique(probs[, 1])))
  currentAgeGroup <- 1
  i <- 1
  den <- sum(meanDat1[1, c(1:5)])
  for(year in age){
    if(year > as.numeric(substr(ageGroups[currentAgeGroup], 4, 5))){
      ageGroupInc1[currentAgeGroup] <- sum(tmp)/sum(tmp2)
      currentAgeGroup <- currentAgeGroup + 1
      tmp <- NULL
      tmp2 <- NULL
    }
    tmp <- c(tmp, newCasesMean1[i])
    tmp2 <- c(tmp2, sum(meanDat1[i, 1:(dim(meanDat1)[2]-2)]))
    i <- i + 1
  }
  ageGroupInc1[length(unique(probs[, 1]))] <- sum(tmp)/sum(tmp2)
  ageGroupInc1 <- ageGroupInc1*100000

  if (!is.null(mort))
  {
    incCCdeath1 <- rep(NA, length(unique(probs[, 1])))
    offset <- as.numeric(substr(probs[1, 1], 1, 2))-1
    for(i in 1:length(ageGroups)){
      tmp <- (as.numeric(substr(ageGroups[i], 1, 2))-offset):(as.numeric(substr(ageGroups[i], 4, 5))-offset)
      num <- mean(CCdeathsMean1[tmp])
      surv <- meanDat1[(as.numeric(substr(ageGroups[i], 1, 2))-offset):(as.numeric(substr(ageGroups[i], 4, 5))-offset), 1:10]
      den <- mean(apply(surv, 1, sum))
      incCCdeath1[i] <- num/den
    }
    incCCdeath1<-incCCdeath1*100000
  }

  f <- which(ageGroups==as.character(prev[1, 1]))
  l <- which(ageGroups==as.character(prev[dim(prev)[1], 1]))
  diff.matrix <- cbind(prev, ageGroupPrev1_pr[f:l], inc, ageGroupInc1[f:l])
  eucl.dist <- vapply(1:dim(diff.matrix)[1], function(i){desv.prev <- ifelse(diff.matrix[i, 2]!=0, abs(diff.matrix[i, 2] - diff.matrix[i,
3])/diff.matrix[i, 2], 0)
  desv.inc  <- ifelse(diff.matrix[i, 5]!=0, abs(diff.matrix[i, 5] - diff.matrix[i, 6])/diff.matrix[i, 5], 0)
  return(desv.prev + desv.inc)}, FUN.VALUE = 1)
  if (n <= keep)
  {
    if(is.null(probs.def[[n]]))
    {
      probs.def[[n]]   <- probs.prova
```

```
      dists[n]        <- sum(eucl.dist[!is.nan(eucl.dist) & eucl.dist!="Inf"], na.rm=T)
      prevalences[[n]] <- ageGroupPrev1_pr
      incidences[[n]]  <- ageGroupInc1
      if (!is.null(mort)) mortality[[n]] <- incCCdeath1
    }
  }
  if (sum(eucl.dist[!is.nan(eucl.dist) & eucl.dist!="Inf"], na.rm=T) != 0)
  {
    if (sum(eucl.dist[!is.nan(eucl.dist) & eucl.dist!="Inf"], na.rm=T) < max(dists, na.rm=T))
    {
      probs.def[[which(dists==max(dists, na.rm=T))[1]]] <- probs.prova
      prevalences[[which(dists==max(dists, na.rm=T))[1]]] <- ageGroupPrev1_pr
      incidences[[which(dists==max(dists, na.rm=T))[1]]] <- ageGroupInc1
      if (!is.null(mort)) mortality[[which(dists==max(dists, na.rm=T))[1]]] <- incCCdeath1
      dists[which(dists==max(dists, na.rm=T))[1]] <- sum(eucl.dist[!is.nan(eucl.dist) & eucl.dist!="Inf"], na.rm=T)
    }
  }
  n <- n + 1
 }
 return(probs.def)
}


NHvalida <- function(probs, prev, inc, mort=NULL, N=500, keep=20, p.change, opt=FALSE)
{
 if (dev.cur() != 1) dev.off()
 p.change <- p.change/100
 if (N < keep) stop("Number of matrices to keep should be lower than the number of simulations")
 if (keep < 2) stop("The minimum is to perform 2 simulations")
 if (!all(prev[, 1] == inc[, 1])) stop("Age groups in reference prevalences/incidences should be equal")
 if (opt==TRUE)
 {
  res <- vector()
  pars <- as.vector(t(probs[, 2:dim(probs)[2]]))
  pars <- pars[pars != 0 & pars != 1]
  res2 <- optim(par=pars, eucl.distance, prob=probs, prev=prev, inc=inc,
          control=list(maxit=100000))
  res  <- res2$par
  probs.optim <- probs
  k <- 1
  for (i in 1:dim(probs)[1])
  {
   for (j in 2:dim(probs)[2])
   {
    if (probs[i, j] == 0 | probs[i, j] == 1)
    {
     probs.optim[i, j] <- probs[i, j]
    }else{
     probs.optim[i, j] <- res[k]
     k <- k + 1
    }
   }
  }
  probs      <- probs.optim
  probs2     <- cbind(probs[, 1], t(apply(probs[, 2:dim(probs)[2]], 1, function(x)(x/(sum(x)))))) ### Normalize each row probability to sum 1
  probs2     <- as.data.frame(probs2)
  probs2[, 1] <- probs[, 1]
  colnames(probs2)[1] <- colnames(probs)[1]
  probs      <- probs2
 }
 probs.def   <- vector("list", keep)
 prevalences <- vector("list", keep)
 incidences  <- vector("list", keep)
 mortality   <- vector("list", keep)
 dists       <- vector()
 prevalence1 <- function(x){
  num <- x[2]
  den <- sum(x[1:10])
  return(num/den)
 }
```

```
incidence1 <- function(i, x, newCasesMean1){
  num <- newCasesMean1[i]
  den <- sum(x[i, c(1:10)])
  num/den
}

n <- 1
while(n <= N)
{
  probs.prova <- matrix(nrow=nrow(probs), ncol=ncol(probs))
  probs.prova[, 1] <- probs[, 1]
  for (i in 1:nrow(probs))
  {
    for (j in 2:ncol(probs))
    {
      if (probs[i, j] != 0 & probs[i, j] != 1)
      {
        probs.prova[i, j] <- sample(rpert(5000, min=max(as.numeric(probs[i, j])*(1-p.change), 0), mode=as.numeric(probs[i, j]),
                                  max=min(c(as.numeric(probs[i, j])*(1+p.change), 1))), 1)
      }else{
        probs.prova[i, j] <- probs[i, j]
      }
    }
  }

  ### Standardize all rows to sum 1
  probs.prova <- cbind(probs.prova[, 1], t(apply(probs.prova[, 2:dim(probs.prova)[2]], 1, function(x)(x/(sum(x))))))
  probs.prova <- as.data.frame(probs.prova)
  probs.prova[, 1] <- probs[, 1]
  cat("Step: ", n, "\n")
  res <- simCohort(probs1=probs.prova, Nsim = 5,
                figoSymProb = c(.11, .23, .66, .9), ### Probability to develop symptoms in FIGO states
                vaccinePrice.md = 0, vaccinePrice.nmd = 0, vaccinePrice.i = 0, screenPrice.md = 0, screenPrice.nmd = 0, screenPrice.i = 0,
                costCoefs.md = c(0, 0, 248.2, 1461.0, 1461.0, 5417.3, 12136.3, 22583.6, 33222.5, 0, 0, 0),
                costCoefs.nmd = c(0, 0, 79.5, 189.6, 189.6, 214.0, 214.0, 214.0, 214.0, 0, 0, 0),
                costCoefs.i = c(0, 0, 0, 0, 0, 0, 0, 0, 0, 0, 0, 0),
                vaccineCov = 0,
                utilityCoefs = c(1, 1, 0.987, 0.87, 0.87, 0.76, 0.67, 0.67, 0.67, 0.938, 0, 0),
                screenProbs = c(0, 0, 1, 1, 1, 0.9688, 0.9066, 0.7064, 0.3986, 0, 0, 0), ndoses = 0)

  tmp <- lapply(as.list(unique(res$age)), function(i, dat){
    aux <- res[res$age == i, ]
    apply(aux[, 1:(ncol(aux)-2)], 2, mean)
  }, dat = res)
  meanDat1 <- as.data.frame(t(as.matrix(as.data.frame(tmp))))
  rownames(meanDat1) <- NULL

  totPrev1 <- apply(meanDat1, 1, prevalence1)

  newCasesMean1 <- apply(attr(res, "newCases"), 2, mean)

  names(newCasesMean1) <- 1:length(newCasesMean1)
  totInc1 <- unlist(lapply(as.list(1:nrow(meanDat1)), incidence1,
                  x = meanDat1, newCasesMean1 = newCasesMean1))

  CCdeathsMean1 <- apply(attr(res, "CCdeaths"), 2, mean)

  age  <- as.numeric(substr(probs[1, 1], 1, 2)):as.numeric(substr(probs[dim(probs)[1], 1], 4, 5))
  ages <- as.numeric(substr(prev[1, 1], 1, 2)):as.numeric(substr(prev[dim(prev)[1], 1], 4, 5))
  HNObsPrev <- approx(prev[, 2], n=length(ages))$y

  tmp1 <- NULL
  tmp2 <- NULL
  ageGroupPrev1_pr <- rep(NA, length(unique(probs[, 1])))
  currentAgeGroup <- 1
  i <- 1
  ageGroups <- unique(probs[, 1])
  for(year in age){
    if(year > as.numeric(substr(ageGroups[currentAgeGroup], 4, 5))){
      ageGroupPrev1_pr[currentAgeGroup] <- sum(tmp1)/sum(tmp2)
```

```
      currentAgeGroup <- currentAgeGroup + 1
      tmp1 <- NULL
      tmp2 <- NULL
    }
    tmp1 <- c(tmp1, meanDat1[i, 2])
    tmp2 <- c(tmp2, sum(meanDat1[i, 1:(dim(meanDat1)[2]-2)]))
    i <- i + 1
  }
  ageGroupPrev1_pr[length(unique(probs[, 1]))] <- sum(tmp1)/sum(tmp2)
  ageGroupNames <- unique(probs[, 1])

  tmp <- NULL
  tmp2 <- NULL
  ageGroupInc1 <- rep(NA, length(unique(probs[, 1])))
  currentAgeGroup <- 1
  i <- 1
  den <- sum(meanDat1[1, c(1:5)])
  for(year in age){
    if(year > as.numeric(substr(ageGroups[currentAgeGroup], 4, 5))){
      ageGroupInc1[currentAgeGroup] <- sum(tmp)/sum(tmp2)
      currentAgeGroup <- currentAgeGroup + 1
      tmp <- NULL
      tmp2 <- NULL
    }
    tmp <- c(tmp, newCasesMean1[i])
    tmp2 <- c(tmp2, sum(meanDat1[i, 1:(dim(meanDat1)[2]-2)]))
    i <- i + 1
  }
  ageGroupInc1[length(unique(probs[, 1]))] <- sum(tmp)/sum(tmp2)
  ageGroupInc1 <- ageGroupInc1*100000

  if (!is.null(mort))
  {
    incCCdeath1 <- rep(NA, length(unique(probs[, 1])))
    offset <- as.numeric(substr(probs[1, 1], 1, 2))-1
    for(i in 1:length(ageGroups)){
      tmp <- (as.numeric(substr(ageGroups[i], 1, 2))-offset):(as.numeric(substr(ageGroups[i], 4, 5))-offset)
      num <- mean(CCdeathsMean1[tmp])
      surv <- meanDat1[(as.numeric(substr(ageGroups[i], 1, 2))-offset):(as.numeric(substr(ageGroups[i], 4, 5))-offset), 1:10]
      den <- mean(apply(surv, 1, sum))
      incCCdeath1[i] <- num/den
    }
    incCCdeath1<-incCCdeath1*100000
  }

  f <- which(ageGroups==as.character(prev[1, 1]))
  l <- which(ageGroups==as.character(prev[dim(prev)[1], 1]))
  diff.matrix <- cbind(prev, ageGroupPrev1_pr[f:l], inc, ageGroupInc1[f:l])
  eucl.dist <- vapply(1:dim(diff.matrix)[1], function(i){desv.prev <- ifelse(diff.matrix[i, 2]!=0, abs(diff.matrix[i, 2] - diff.matrix[i, 
3])/diff.matrix[i, 2], 0)
                      desv.inc  <- ifelse(diff.matrix[i, 5]!=0, abs(diff.matrix[i, 5] - diff.matrix[i, 6])/diff.matrix[i, 5], 0)
  return(desv.prev + desv.inc)}, FUN.VALUE = 1)
  if (n <= keep)
  {
    if(is.null(probs.def[[n]]))
    {
      probs.def[[n]]   <- probs.prova
      dists[n]         <- sum(eucl.dist)
      prevalences[[n]] <- ageGroupPrev1_pr
      incidences[[n]]  <- ageGroupInc1
      if (!is.null(mort)) mortality[[n]] <- incCCdeath1
    }
  }
  if (sum(eucl.dist) != 0)
  {
    if (sum(eucl.dist) < max(dists, na.rm=T))
    {
      probs.def[[which(dists==max(dists, na.rm=T))[1]]] <- probs.prova
      prevalences[[which(dists==max(dists, na.rm=T))[1]]] <- ageGroupPrev1_pr
      incidences[[which(dists==max(dists, na.rm=T))[1]]] <- ageGroupInc1
```

```
      if (!is.null(mort)) mortality[[which(dists==max(dists, na.rm=T))[1]]] <- incCCdeath1
      dists[which(dists==max(dists, na.rm=T))[1]] <- sum(eucl.dist)
    }
   }
   n <- n + 1
  }

  #### plot the graph with prevalences from selected matrices vs real prevalence
  if (!is.null(mort)) par(las = 2, mfrow = c(3, 1))
  if (is.null(mort))  par(las = 2, mfrow = c(2, 1))
  maxim <- max(sapply(prevalences, max), prev[, 2])
  plot(1:length(prevalences[[1]]), prevalences[[1]], pch = 20, axes = FALSE,
      xlab = "", ylab = "Prevalence", main = "HPV prevalence per age group",
      ylim = c(0, maxim+0.05), col="grey")
  lines(1:length(prevalences[[1]]), prevalences[[1]], col="grey", lwd = 2)
  for (i in 2:length(prevalences)) ### length(prevalences) should be equal to 'keep' parameter
  {
    lines(1:length(prevalences[[i]]), prevalences[[i]], pch=20, type="p", col="grey")
    lines(1:length(prevalences[[i]]), prevalences[[i]], lwd=2, col="grey")
  }
  lines(f:l, prev[, 2], col = "black", type = "p", pch = 20)
  lines(f:l, prev[, 2], col = "black", lwd = 2)
  axis(2)
  axis(1, labels = ageGroupNames, at = 1:length(ageGroupPrev1_pr))
  legend("topright", c("Model simulations", "Observed prevalence"),
        pch = 20, col = c("grey", "black"), lwd = 2, bty = "n")
  maxim <- max(sapply(incidences, max), inc[, 2])
  plot(1:length(incidences[[1]]), incidences[[1]], pch = 20, axes = FALSE,
      xlab = "", ylab = "Incidence", main = "CC incidence per age group",
      ylim = c(0, maxim+0.05), col="grey")
  lines(1:length(incidences[[1]]), incidences[[1]], lwd = 2, col="grey")
  for (i in 2:length(incidences)) ### length(incidences) should be equal to 'keep' parameter
  {
    lines(1:length(incidences[[i]]), incidences[[i]], pch=20, type="p", col="grey")
    lines(1:length(incidences[[i]]), incidences[[i]], lwd=2, col="grey")
  }
  lines(f:l, inc[, 2], col = "black", type = "p", pch = 20)
  lines(f:l, inc[, 2], col = "black", lwd = 2)
  axis(2)
  axis(1, labels = ageGroupNames, at = 1:length(ageGroupInc1))
  legend("topleft", c("Model simulations", "Observed incidence"),
        pch = 20, col = c("grey", "black"), lwd = 2, bty = "n")
  if (!is.null(mort))
  {
    maxim <- max(sapply(mortality, max), mort[, 2])
    plot(1:length(mortality[[1]]), mortality[[1]], pch = 20, axes = FALSE,
        xlab = "", ylab = "Mortality", main = "CC mortality per age group",
        ylim = c(0, maxim+0.05), col="grey")
    lines(1:length(mortality[[1]]), mortality[[1]], col="grey", lwd = 2)
    for (i in 2:length(mortality)) ### length(mortality) should be equal to 'keep' parameter
    {
      lines(1:length(mortality[[i]]), mortality[[i]], pch=20, type="p", col="grey")
      lines(1:length(mortality[[i]]), mortality[[i]], lwd=2, col="grey")
    }
    lines(f:l, mort[, 2], col = "black", type = "p", pch = 20)
    lines(f:l, mort[, 2], col = "black", lwd = 2)
    axis(2)
    axis(1, labels = ageGroupNames, at = 1:length(incCCdeath1))
    legend("topleft", c("Model simulations", "Observed mortality"),
          pch = 20, col = c("grey", "black"), lwd = 2, bty = "n")
  }
  return(probs.def)
}


eucl.distance <- function(pars, prob, prev, inc)
{
  prevalence1 <- function(x){
    num <- x[2]
    den <- sum(x[1:10])
```

```
  return(num/den)
}

incidence1 <- function(i, x, newCasesMean1){
  num <- newCasesMean1[i]
  den <- sum(x[i, c(1:10)])
  num/den
}

prob2 <- prob
k <- 1
for (i in 1:dim(prob)[1])
{
  for (j in 2:dim(prob)[2])
  {
    if (prob[i, j] == 0 | prob[i, j] == 1 | is.nan(pars[k]))
    {
      prob2[i, j] <- prob[i, j]
    }else{
      prob2[i, j] <- pars[k]
      k <- k + 1
    }
  }
}
prob2 <- cbind(prob2[, 1], t(apply(prob2[, 2:dim(prob2)[2]], 1, function(x)(x/(sum(x))))))
prob2 <- as.data.frame(prob2)
prob2[, 1] <- prob[, 1]

for (i in 1:dim(prob2)[1])
{
  for (j in 2:dim(prob2)[2])
  {
    if (is.nan(prob2[i, j])) prob2[i, j] <- prob[i, j]
  }
}
res <- simCohort(probs1=prob2, Nsim = 5, figoSymProb = c(.11, .23, .66, .9), ### Probability to develop symptoms in FIGO states
                 vaccinePrice.md = 0, vaccinePrice.nmd = 0, vaccinePrice.i = 0, screenPrice.md = 0, screenPrice.nmd = 0, screenPrice.i = 0,
                 costCoefs.md = c(0, 0, 248.2, 1461.0, 1461.0, 5417.3, 12136.3, 22583.6, 33222.5, 0, 0, 0),
                 costCoefs.nmd = c(0, 0, 79.5, 189.6, 189.6, 214.0, 214.0, 214.0, 214.0, 0, 0, 0),
                 costCoefs.i = c(0, 0, 0, 0, 0, 0, 0, 0, 0, 0, 0, 0),
                 vaccineCov = 0, utilityCoefs = c(1, 1, 0.987, 0.87, 0.87, 0.76, 0.67, 0.67, 0.67, 0.938, 0, 0),
                 screenProbs = c(0, 0, 1, 1, 1, 0.9688, 0.9066, 0.7064, 0.3986, 0, 0, 0), ndoses = 0)
tmp <- lapply(as.list(unique(res$age)), function(i, dat){
  aux <- res[res$age == i, ]
  apply(aux[, 1:(ncol(aux)-2)], 2, mean)
}, dat = res)
meanDat1 <- as.data.frame(t(as.matrix(as.data.frame(tmp))))
rownames(meanDat1) <- NULL
totPrev1 <- apply(meanDat1, 1, prevalence1)

newCasesMean1 <- apply(attr(res, "newCases"), 2, mean)

names(newCasesMean1) <- 1:length(newCasesMean1)

totInc1 <- unlist(lapply(as.list(1:nrow(meanDat1)), incidence1,
                   x = meanDat1, newCasesMean1 = newCasesMean1))

age  <- as.numeric(substr(prob[1, 1], 1, 2)):as.numeric(substr(prob[dim(prob)[1], 1], 4, 5))
ages <- as.numeric(substr(prev[1, 1], 1, 2)):as.numeric(substr(prev[dim(prev)[1], 1], 4, 5))
HNObsPrev <- approx(prev[, 2], n=length(ages))$y

tmp1 <- NULL
tmp2 <- NULL
ageGroupPrev1_pr <- rep(NA, length(unique(unique(prob[, 1]))))
currentAgeGroup  <- 1
i <- 1
ageGroups <- unique(prob[, 1])
for(year in age){
  if(year > as.numeric(substr(ageGroups[currentAgeGroup], 4, 5))){
    ageGroupPrev1_pr[currentAgeGroup] <- sum(tmp1)/sum(tmp2)
```

```
      currentAgeGroup <- currentAgeGroup + 1
      tmp1 <- NULL
      tmp2 <- NULL
    }
    tmp1 <- c(tmp1, meanDat1[i, 2])
    tmp2 <- c(tmp2, sum(meanDat1[i, 1:(dim(meanDat1)[2]-2)]))
    i <- i + 1
  }
  ageGroupPrev1_pr[length(unique(prob[, 1]))] <- sum(tmp1)/sum(tmp2)

  ageGroupNames <- unique(prob[, 1])

  tmp <- NULL
  tmp2 <- NULL
  ageGroupInc1 <- rep(NA, length(unique(unique(prob[, 1]))))
  currentAgeGroup <- 1
  i <- 1
  den <- sum(meanDat1[1, c(1:10)])
  for(year in age){
    if(year > as.numeric(substr(ageGroups[currentAgeGroup], 4, 5))){
      ageGroupInc1[currentAgeGroup] <- sum(tmp)/sum(tmp2)
      currentAgeGroup <- currentAgeGroup + 1
      tmp <- NULL
      tmp2 <- NULL
    }
    tmp <- c(tmp, newCasesMean1[i])
    tmp2 <- c(tmp2, sum(meanDat1[i, 1:(dim(meanDat1)[2]-2)]))
    i <- i + 1
  }
  ageGroupInc1[length(unique(prob[, 1]))] <- sum(tmp)/sum(tmp2)
  ageGroupInc1 <- ageGroupInc1*100000

  f <- which(ageGroups==as.character(prev[1, 1]))
  l <- which(ageGroups==as.character(prev[dim(prev)[1], 1]))
  diff.matrix <- cbind(prev, ageGroupPrev1_pr[f:l], inc, ageGroupInc1[f:l])
  desv.prev <- ifelse(diff.matrix[, 2]!=0, abs(diff.matrix[, 2] - diff.matrix[, 3])/diff.matrix[, 2], 0)
  desv.inc  <- ifelse(diff.matrix[, 5]!=0, abs(diff.matrix[, 5] - diff.matrix[, 6])/diff.matrix[, 5], 0)
  eucl.dist <- desv.prev + desv.inc
  return(sum(eucl.dist[!is.nan(eucl.dist) & eucl.dist!="Inf"], na.rm=T))
}

simCohort <- function(probs1, probs2 = NULL, probs3 = NULL, stopYear = 85, stepTime = 1,
                M = 1e5, Nsim = 500, propDiffStrain = 0.70, propNoHPV = 0.0,
                screening = FALSE, screenCoverage = NULL, screenSensi = NULL, screenProbs = NULL,### vaccinePrice is cost per dose, number
of doses in ndoses
                figoSymProb, vaccinePrice.md, vaccinePrice.nmd, vaccinePrice.i, screenPrice.md, screenPrice.nmd, screenPrice.i,
                vaccineCov, costCoefs.md, costCoefs.nmd, ### Direct medical; Direct non medical
                costCoefs.i, utilityCoefs, screenPeriod = 3, seed = 1234, dnaScreening = FALSE,  ### Indirect costs
                dnaScSensi = NULL, dnaScCost.md = NULL, dnaScCost.nmd = NULL, dnaScCost.i = NULL, papScSensi = NULL, dnaScAgeGroups =
NULL,
                newScreenPrice.md, newScreenPrice.nmd, newScreenPrice.i, newScreenCoverage, ScreenType = 0, OpScreen = NULL,
ndoses=0,
                via = FALSE, via.Sensi = NULL, viaCoverage = NULL, viaProbs = NULL, viaPeriod = 0, via.md=0, via.nmd=0, via.i=0) ### VIA
  ### ScreenType = 0 -> No screening
  ### ScreenType = 1 -> Organized screening
  ### ScreenType = 2 -> Opportunistic screening, needs file with proportions (OpScreen)
{
  ll <- as.list(match.call())[-1]     ##
  myfor <- formals(simCohort)         ## formals with default arguments
  for (v in names(myfor)){
    if (!(v %in% names(ll)))
      ll <- append(ll, myfor[v])      ## if arg is missing I add it
  }
  if(!is.null(seed))
  {
    set.seed(seed)
  }else{
    set.seed(1234)
  }
  if (via == TRUE & is.null(via.Sensi)) stop("If VIA scenario is selected, VIA sensitivity should be provided")
```

```
if ((vaccinePrice.md > 0 | vaccinePrice.nmd > 0 | vaccinePrice.i > 0) & ndoses == 0) stop("Number of vaccine doses should be at least 1")
if (vaccinePrice.md == 0 & vaccinePrice.nmd == 0 & vaccinePrice.i == 0 & ndoses > 0) stop("Vaccine price per dose should be more than 0")
if (screening == FALSE & ScreenType != 0) stop("If no screening, 'ScreenType' should be 0")
if (screening == TRUE  & ScreenType == 0) stop("If screening, 'ScreenType' should be 1 (organized) or 2 (opportunistic)")
if (ScreenType == 2)
{
  if (is.null(OpScreen)) stop("File with oportunistic screening details needed")
  if (sum(OpScreen[, 2]) != 100) stop("Wrong proportions in oportunistic screening details file")
}
totNumSteps <-(stopYear-as.numeric(substr(probs1[, 1], 1, 2))[1])/stepTime
ageGroups <- unique(data.frame(Start=as.numeric(substr(probs1[, 1], 1, 2)), End=as.numeric(substr(probs1[, 1], 4, 5))))
probs1 <- split(probs1[, 2:dim(probs1)[2]], probs1[, 1])
if (!is.null(probs2)) probs2 <- split(probs2[, 2:dim(probs2)[2]], probs2[, 1])
if (!is.null(probs3)) probs3 <- split(probs3[, 2:dim(probs3)[2]], probs3[, 1])
K <- length(probs1)    ### Age groups
P <- ncol(probs1[[1]]) ### Health states
res <- list()
func <- function(sim){
  if(!is.null(probs2)){
    if(is.null(probs3))
      stop("If 'probs2' is provided 'probs3' must also be provided")
    propNormal <- 1 - propDiffStrain - propNoHPV
    probs <- lapply(1:length(probs1), function(i, probs1, probs2, probs3, prop1, prop2, prop3)
      prop1*probs1[[i]] + prop2*probs2[[i]] + prop3*probs3[[i]],
      probs1 = probs1, probs2 = probs2, probs3 = probs3,
      prop1 = propNormal, prop2 = propDiffStrain,
      prop3 = propNoHPV)
  }
  else
    probs <- probs1
  cumProbs <- lapply(probs, function(x)
    t(apply(x, 1, cumsum)))
  hpvProbK <- lapply(probs2, function(x) unlist(x[1, ]))
  hpvProbKCum <- lapply(hpvProbK, cumsum)
  if(screening)
    if(is.null(screenCoverage) || is.null(screenSensi) || is.null(screenProbs))
      stop("If screening scenario is selected 'screenCoverage', 'screenSensi' and 'screenProbs' must be provided")
  if(dnaScreening){
    if(!screening)
      stop("'dnaScreening = TRUE' is only available when 'screening = TRUE'")
    if(is.null(dnaScSensi)||is.null(dnaScCost.md)||is.null(dnaScCost.nmd)||is.null(dnaScCost.i)||is.null(papScSensi)||
      is.null(dnaScAgeGroups))
      stop("'dnaScSensi', 'dnaScCost.md', 'dnaScCost.nmd', 'dnaScCost.i', 'papScSensi' and 'dnaScAgeGroups' must be provided when
'dnaScreening = TRUE'")
  }
  if(via)
  {
    if (is.null(via.md) || is.null(via.nmd) || is.null(via.i)) stop("'via.md', 'via.nmd' and 'via.i' must be provided when 'via = TRUE'")
  }
  stageNi <- rep(0, P)
  names(stageNi) <- names(probs[[1]])
  stageN <- rep(list(stageNi), totNumSteps)
  attr(stageN[[1]], "newCases")          <- 0 # No new cases in the first step!
  attr(stageN[[1]], "newCin1Cases")      <- 0
  attr(stageN[[1]], "newCin2Cases")      <- 0
  attr(stageN[[1]], "newCin3Cases")      <- 0
  attr(stageN[[1]], "newSurvivalCases") <- 0
  attr(stageN[[1]], "CCdeaths")          <- 0
  attr(stageN[[1]], "Otherdeaths")       <- 0
  attr(stageN[[1]], "utility")           <- M
  attr(stageN[[1]], "probs_k")           <- rep(0, 9)
  attr(stageN[[1]], "probs_u")           <- rep(0, 9)
  attr(stageN[[1]], "nCito")             <- 0
  attr(stageN[[1]], "nVPH")              <- 0
  stageN[[1]][1]                         <- attr(stageN[[1]], "probs_u")[1] <- M
  if(!is.null(probs2) && !is.null(probs3)) # Assign to first step the vaccine cost in vaccination scenario
  {
    attr(stageN[[1]], "md_cost")  <- vaccineCov*vaccinePrice.md*M*ndoses
    attr(stageN[[1]], "nmd_cost") <- vaccineCov*vaccinePrice.nmd*M*ndoses
    attr(stageN[[1]], "i_cost")   <- vaccineCov*vaccinePrice.i*M*ndoses
```

```
  }else{
   attr(stageN[[1]], "md_cost")  <- 0
   attr(stageN[[1]], "nmd_cost") <- 0
   attr(stageN[[1]], "i_cost")   <- 0
  }
  currentAge <- ageGroups[1,1]
  currentAgeGroup <- 1 # Age group
  screenCount <- 1
  viaCount    <- 1
  for(i in 1:(totNumSteps-1)){ # Step loop
   if(currentAge > ageGroups$End[currentAgeGroup])
     currentAgeGroup <- currentAgeGroup + 1 # Change the age group if pertinent
   J <- which(stageN[[i]] > 0)
   stageN[[i+1]] <- stageN[[i]] # Initialize next step
   attr(stageN[[i+1]], "nCito") <- 0
   attr(stageN[[i+1]], "nVPH")  <- 0
   attr(stageN[[i+1]], "probs_k") <- attr(stageN[[i]], "probs_k")
   attr(stageN[[i+1]], "probs_u") <- attr(stageN[[i]], "probs_u")
   newSurvivalCases <- 0
   cost.md  <- 0
   cost.nmd <- 0
   cost.i   <- 0
   for(j in J){ # Stage loop
    if(j%in%1:9){ # CIN or FIGO
      nU <- attr(stageN[[i]], "probs_u")[j]
      nK <- attr(stageN[[i]], "probs_k")[j]
      pU <- runif(nU)
      pK <- runif(nK)
      qU <- findInterval(pU, unlist(cumProbs[[currentAgeGroup]][j,])) + 1
      qK <- findInterval(pK,
                  unlist(cumProbs[[currentAgeGroup]][j,])) + 1
      outU <- sum(qU != j)
      outK <- sum(qK != j)
      attr(stageN[[i+1]], "probs_u")[j] <-
        attr(stageN[[i+1]], "probs_u")[j] - outU
      attr(stageN[[i+1]], "probs_k")[j] <-
        attr(stageN[[i+1]], "probs_k")[j] - outK
      if(j == 2)
        attr(stageN[[i+1]], "newCin1Cases") <-
        sum(qU == 3) + sum(qK == 3)
      if(j == 3)
        attr(stageN[[i+1]], "newCin2Cases") <-
        sum(qU == 4) + sum(qK == 4)
      if(j == 4)
        attr(stageN[[i+1]], "newCin3Cases") <-
        sum(qU == 5) + sum(qK == 5)
      if(j == 5) # Store the number of subjects arriving to FIGO.I from CIN3 (new-cases)
        attr(stageN[[i+1]], "newCases") <- sum(qU == 6) + sum(qK == 6)
      for(l in union(unique(qU), unique(qK))){
        if(l != j){
          if(l%in%10:12)
            stageN[[i+1]][l] <- stageN[[i+1]][l] + sum(qU==l) + sum(qK==l)
          else{
            attr(stageN[[i+1]], "probs_u")[l] <-
              attr(stageN[[i+1]], "probs_u")[l] + sum(qU==l)
            attr(stageN[[i+1]], "probs_k")[l] <-
              attr(stageN[[i+1]], "probs_k")[l] + sum(qK==l)
          }
        }
      }
      if((screening)&&(screenCount%%screenPeriod == 0) && ScreenType == 1){## ORGANIZED SCREENING
        pU <- runif(attr(stageN[[i+1]], "probs_u")[j])
        pK <- runif(attr(stageN[[i+1]], "probs_k")[j])
        nScreenedU <- sum(pU < screenCoverage[currentAgeGroup])
        nScreenedK <- sum(pK < screenCoverage[currentAgeGroup])
        if(dnaScreening && currentAgeGroup%in%dnaScAgeGroups){
          cost.md <- cost.md + nScreenedU*dnaScCost.md
          cost.nmd <- cost.nmd + nScreenedU*dnaScCost.nmd
          cost.i <- cost.i + nScreenedU*dnaScCost.i
          p2 <- runif(nScreenedU)
```

```
  attr(stageN[[i+1]], "nVPH") <- ifelse(!is.null(attr(stageN[[i+1]], "nVPH")), attr(stageN[[i+1]], "nVPH")+nScreenedU+nScreenedK,
                                nScreenedU+nScreenedK)
  nDetected <- sum(p2 < dnaScSensi[j])
  attr(stageN[[i+1]], "nCito") <- ifelse(!is.null(attr(stageN[[i+1]], "nCito")), attr(stageN[[i+1]], "nCito")+nDetected,
                                nDetected)
  p22 <- runif(nDetected)
  nPapDetected <- sum(p22 < papScSensi[j])
  cost.md <- cost.md + nDetected*screenPrice.md
  cost.nmd <- cost.nmd + nDetected*screenPrice.nmd
  cost.i <- cost.i + nDetected*screenPrice.i
  cost.md <- cost.md + nPapDetected*costCoefs.md[j]
  cost.nmd <- cost.nmd + nPapDetected*costCoefs.nmd[j]
  cost.i <- cost.i + nPapDetected*costCoefs.i[j]
  p3 <- runif(nPapDetected)
  nRecovered <- sum(p3 < screenProbs[j])
  attr(stageN[[i+1]], "probs_u")[j] <-
   attr(stageN[[i+1]], "probs_u")[j] - nPapDetected
  attr(stageN[[i+1]], "probs_k")[j] <-
   attr(stageN[[i+1]], "probs_k")[j] + nPapDetected - nRecovered
  if(j <= 5)
   attr(stageN[[i+1]], "probs_u")[1] <-
   attr(stageN[[i+1]], "probs_u")[1] + nRecovered
  else{
   stageN[[i+1]][10] <- stageN[[i+1]][10] + nRecovered
   newSurvivalCases <- newSurvivalCases + nRecovered
  }
 }
 else{
  cost.md <- cost.md + nScreenedU*screenPrice.md
  cost.nmd <- cost.nmd + nScreenedU*screenPrice.nmd
  cost.i <- cost.i + nScreenedU*screenPrice.i
  p2 <- runif(nScreenedU)
  attr(stageN[[i+1]], "nVPH") <- 0
  attr(stageN[[i+1]], "nCito") <- ifelse(!is.null(attr(stageN[[i+1]], "nCito")), attr(stageN[[i+1]], "nCito")+nScreenedU+nScreenedK,
                                nScreenedU+nScreenedK)
  nDetected <- sum(p2 < screenSensi[j])
  cost.md <- cost.md + nDetected*costCoefs.md[j]
  cost.nmd <- cost.nmd + nDetected*costCoefs.nmd[j]
  cost.i <- cost.i + nDetected*costCoefs.i[j]
  p3 <- runif(nDetected)
  nRecovered <- sum(p3 < screenProbs[j])
  attr(stageN[[i+1]], "probs_u")[j] <-
   attr(stageN[[i+1]], "probs_u")[j] - nDetected
  attr(stageN[[i+1]], "probs_k")[j] <-
   attr(stageN[[i+1]], "probs_k")[j] + nDetected - nRecovered
  if(j <= 5)
   attr(stageN[[i+1]], "probs_u")[1] <-
   attr(stageN[[i+1]], "probs_u")[1] + nRecovered
  else{
   stageN[[i+1]][10] <- stageN[[i+1]][10] + nRecovered
   newSurvivalCases <- newSurvivalCases + nRecovered
  }
 }
 cost.md <- cost.md + nScreenedK*newScreenPrice.md
 cost.nmd <- cost.nmd + nScreenedK*newScreenPrice.nmd
 cost.i <- cost.i + nScreenedK*newScreenPrice.i
}
if((screening) && ScreenType == 2){## OPPORTUNISTIC SCREENING
 for (k in 1:nrow(OpScreen))
 {
  screenPeriod <- OpScreen[k, 1]
  if (screenCount%%screenPeriod == 0 & OpScreen[k, 2] != 0)
  {
   pU <- runif(attr(stageN[[i+1]], "probs_u")[j])
   pK <- runif(attr(stageN[[i+1]], "probs_k")[j])
   nScreenedU <- sum(pU < screenCoverage[currentAgeGroup])*OpScreen[k, 2]/100
   nScreenedK <- sum(pK < screenCoverage[currentAgeGroup])*OpScreen[k, 2]/100
   if(dnaScreening && currentAgeGroup%in%dnaScAgeGroups){
    cost.md <- cost.md + nScreenedU*dnaScCost.md
    cost.nmd <- cost.nmd + nScreenedU*dnaScCost.nmd
```

```
          cost.i <- cost.i + nScreenedU*dnaScCost.i
          p2 <- runif(nScreenedU)
          nDetected <- sum(p2 < dnaScSensi[j])
          attr(stageN[[i+1]], "nVPH") <- ifelse(!is.null(attr(stageN[[i+1]], "nVPH")), attr(stageN[[i+1]], "nVPH")+nScreenedU+nScreenedK,
                                       nScreenedU+nScreenedK)
          attr(stageN[[i+1]], "nCito") <- ifelse(!is.null(attr(stageN[[i+1]], "nCito")), attr(stageN[[i+1]], "nCito")+nDetected,
                                        nDetected)
          p22 <- runif(nDetected)
          nPapDetected <- sum(p22 < papScSensi[j])
          cost.md <- cost.md + nDetected*screenPrice.md
          cost.nmd <- cost.nmd + nDetected*screenPrice.nmd
          cost.i <- cost.i + nDetected*screenPrice.i
          cost.md <- cost.md + nPapDetected*costCoefs.md[j]
          cost.nmd <- cost.nmd + nPapDetected*costCoefs.nmd[j]
          cost.i <- cost.i + nPapDetected*costCoefs.i[j]
          p3 <- runif(nPapDetected)
          nRecovered <- sum(p3 < screenProbs[j])
          attr(stageN[[i+1]], "probs_u")[j] <-
            attr(stageN[[i+1]], "probs_u")[j] - nPapDetected
          attr(stageN[[i+1]], "probs_k")[j] <-
            attr(stageN[[i+1]], "probs_k")[j] + nPapDetected - nRecovered
          if(j <= 5)
            attr(stageN[[i+1]], "probs_u")[1] <-
              attr(stageN[[i+1]], "probs_u")[1] + nRecovered
          else{
            stageN[[i+1]][10] <- stageN[[i+1]][10] + nRecovered
            newSurvivalCases <- newSurvivalCases + nRecovered
          }
        }
        else{
          cost.md <- cost.md + nScreenedU*screenPrice.md
          cost.nmd <- cost.nmd + nScreenedU*screenPrice.nmd
          cost.i <- cost.i + nScreenedU*screenPrice.i
          p2 <- runif(nScreenedU)
          attr(stageN[[i+1]], "nCito") <- ifelse(!is.null(attr(stageN[[i+1]], "nCito")), attr(stageN[[i+1]], "nCito")+nScreenedU+nScreenedK,
                                        +nScreenedU+nScreenedK)
          attr(stageN[[i+1]], "nVPH") <- 0
          nDetected <- sum(p2 < screenSensi[j])
          cost.md <- cost.md + nDetected*costCoefs.md[j]
          cost.nmd <- cost.nmd + nDetected*costCoefs.nmd[j]
          cost.i <- cost.i + nDetected*costCoefs.i[j]
          p3 <- runif(nDetected)
          nRecovered <- sum(p3 < screenProbs[j])
          attr(stageN[[i+1]], "probs_u")[j] <-
            attr(stageN[[i+1]], "probs_u")[j] - nDetected
          attr(stageN[[i+1]], "probs_k")[j] <-
            attr(stageN[[i+1]], "probs_k")[j] + nDetected - nRecovered
          if(j <= 5)
            attr(stageN[[i+1]], "probs_u")[1] <-
            attr(stageN[[i+1]], "probs_u")[1] + nRecovered
          else{
            stageN[[i+1]][10] <- stageN[[i+1]][10] + nRecovered
            newSurvivalCases <- newSurvivalCases + nRecovered
          }
        }
        cost.md <- cost.md + nScreenedK*newScreenPrice.md
        cost.nmd <- cost.nmd + nScreenedK*newScreenPrice.nmd
        cost.i <- cost.i + nScreenedK*newScreenPrice.i
      }
    }
  }
  if((via)&&(viaCount%%viaPeriod == 0)){## VIA
    pU <- runif(attr(stageN[[i+1]], "probs_u")[j])
    pK <- runif(attr(stageN[[i+1]], "probs_k")[j])
    nScreenedVIAU <- sum(pU < viaCoverage[currentAgeGroup])
    nScreenedVIAK <- sum(pK < viaCoverage[currentAgeGroup])
    cost.md <- cost.md + nScreenedVIAU*via.md
    cost.nmd <- cost.nmd + nScreenedVIAU*via.nmd
    cost.i <- cost.i + nScreenedVIAU*via.i
    p2 <- runif(nScreenedVIAU)
```

```
        nDetected <- sum(p2 < viaSensi[j])
        cost.md <- cost.md + nDetected*costCoefs.md[j]
        cost.nmd <- cost.nmd + nDetected*costCoefs.nmd[j]
        cost.i <- cost.i + nDetected*costCoefs.i[j]
        p3 <- runif(nDetected)
        nRecovered <- sum(p3 < viaProbs[j])
        attr(stageN[[i+1]], "probs_u")[j] <-
          attr(stageN[[i+1]], "probs_u")[j] - nDetected
        attr(stageN[[i+1]], "probs_k")[j] <-
          attr(stageN[[i+1]], "probs_k")[j] + nDetected - nRecovered
        if(j <= 5)
          attr(stageN[[i+1]], "probs_u")[1] <-
            attr(stageN[[i+1]], "probs_u")[1] + nRecovered
        else{
          stageN[[i+1]][10] <- stageN[[i+1]][10] + nRecovered
          newSurvivalCases <- newSurvivalCases + nRecovered
        }
      }
      ## GENERATE SYMPTOMS (COST COMPUTATIONS)
      if(j%in%6:9){
        cost.md <- cost.md + costCoefs.md[j]*attr(stageN[[i+1]], "probs_k")[j]
        cost.nmd <- cost.nmd + costCoefs.nmd[j]*attr(stageN[[i+1]], "probs_k")[j]
        cost.i <- cost.i + costCoefs.i[j]*attr(stageN[[i+1]], "probs_k")[j]
        p <- runif(attr(stageN[[i+1]], "probs_u")[j])
        nSymp <- sum(p < figoSymProb[j - 5])
        cost.md <- cost.md + nSymp*costCoefs.md[j]
        cost.nmd <- cost.nmd + nSymp*costCoefs.nmd[j]
        cost.i <- cost.i + nSymp*costCoefs.i[j]
        p <- runif(nSymp)
        nCured <- sum(p < screenProbs[j])
        attr(stageN[[i+1]], "probs_u")[j] <-
          attr(stageN[[i+1]], "probs_u")[j] - nSymp
        attr(stageN[[i+1]], "probs_k")[j] <-
          attr(stageN[[i+1]], "probs_k")[j] + nSymp - nCured
        stageN[[i+1]][10] <- stageN[[i+1]][10] + nCured
        newSurvivalCases <- newSurvivalCases + nCured
      }
    }
    else{
      p <- runif(stageN[[i]][j]) # Generate Uniform(0,1) random numbers for each individual at the stage
      q <- findInterval(p, unlist(cumProbs[[currentAgeGroup]][j,])) + 1 # Assign the generated numbers to the cumulative probabilities
      out <- sum(q != j) # Number of individuals leaving the stage
      stageN[[i+1]][j] <- stageN[[i+1]][j] - out # Subtract the number of individuals leaving the stage
      for(l in unique(q)){ # Loop for assigning the leaving individuals to their new stage
        if(l!=j){
          if(l%in%3:9)
            attr(stageN[[i+1]], "probs_u")[l-2] <-
              attr(stageN[[i+1]], "probs_u")[l-2] + sum(q == l)
          else
            stageN[[i+1]][l] <- stageN[[i+1]][l] + sum(q == l)
        }
      }
      if(j != 10)
        newSurvivalCases <- newSurvivalCases + sum(q == 10)
      if((screening) && (screenCount%%screenPeriod == 0) && (j == 10) && ScreenType==1){
        p <- runif(stageN[[i+1]][j])
        nScreened <- sum(p < screenCoverage[currentAgeGroup])
        if(dnaScreening && currentAgeGroup%in%dnaScAgeGroups){
          cost.md <- cost.md + nScreened*dnaScCost.md
          cost.nmd <- cost.nmd + nScreened*dnaScCost.nmd
          cost.i <- cost.i + nScreened*dnaScCost.i
          cost.md <- cost.md + nScreened*screenPrice.md
          cost.nmd <- cost.nmd + nScreened*screenPrice.nmd
          cost.i <- cost.i + nScreened*screenPrice.i
          attr(stageN[[i+1]], "CCdeaths") <- stageN[[i+1]][11] - stageN[[i]][11]
          names(attr(stageN[[i+1]], "CCdeaths")) <- NULL
          attr(stageN[[i+1]], "Otherdeaths") <- stageN[[i+1]][12] - stageN[[i]][12]
          names(attr(stageN[[i+1]], "Otherdeaths")) <- NULL
        }
      }
```

```
    if((screening) && (j == 10) && ScreenType==2){
     for (k in 1:nrow(OpScreen))
     {
      screenPeriod <- OpScreen[k, 1]
      if (screenCount%%screenPeriod == 0 & OpScreen[k, 2] != 0)
      {
       p <- runif(stageN[[i+1]][j])
       nScreened <- sum(p < screenCoverage[currentAgeGroup])*OpScreen[k, 2]/100
       if(dnaScreening && currentAgeGroup%in%dnaScAgeGroups){
        cost.md <- cost.md + nScreened*dnaScCost.md
        cost.nmd <- cost.nmd + nScreened*dnaScCost.nmd
        cost.i <- cost.i + nScreened*dnaScCost.i
        cost.md <- cost.md + nScreened*screenPrice.md
        cost.nmd <- cost.nmd + nScreened*screenPrice.nmd
        cost.i <- cost.i + nScreened*screenPrice.i
        attr(stageN[[i+1]], "CCdeaths") <- stageN[[i+1]][11] - stageN[[i]][11]
        names(attr(stageN[[i+1]], "CCdeaths")) <- NULL
        attr(stageN[[i+1]], "Otherdeaths") <- stageN[[i+1]][12] - stageN[[i]][12]
        names(attr(stageN[[i+1]], "Otherdeaths")) <- NULL
       }
      }
     }
    }
   }
  }
  for(j in 1:9)
   stageN[[i+1]][j] <- attr(stageN[[i+1]], "probs_u")[j] +
   attr(stageN[[i+1]], "probs_k")[j]
  attr(stageN[[i+1]], "newSurvivalCases") <- newSurvivalCases
  attr(stageN[[i+1]], "md_cost") <- cost.md
  attr(stageN[[i+1]], "nmd_cost") <- cost.nmd
  attr(stageN[[i+1]], "i_cost") <- cost.i
  names(stageN)[[i]] <- currentAge
  currentAge <- currentAge + stepTime
  attr(stageN[[i+1]], "utility") <- utilityCoefs%*%stageN[[i+1]]
  attr(stageN[[i+1]], "CCdeaths") <- stageN[[i+1]][11] - stageN[[i]][11]
  names(attr(stageN[[i+1]], "CCdeaths")) <- NULL
  attr(stageN[[i+1]], "Otherdeaths") <- stageN[[i+1]][12] - stageN[[i]][12]
  names(attr(stageN[[i+1]], "Otherdeaths")) <- NULL
  screenCount <- screenCount + 1
  viaCount    <- viaCount + 1
 }
 names(stageN)[[length(stageN)]] <- currentAge
 return(stageN)
}
res <- lapply(seq(1,Nsim,1), func)
listDat <- lapply(res, function(x){
 y <- as.data.frame(t(as.matrix(as.data.frame(x))))
 aux <- unlist(lapply(x, function(x) attr(x, "newCases")))
 attr(y, "newCases") <- aux
 aux <- unlist(lapply(x, function(x) attr(x, "newCin1Cases")))
 attr(y, "newCin1Cases") <- aux
 aux <- unlist(lapply(x, function(x) attr(x, "newCin2Cases")))
 attr(y, "newCin2Cases") <- aux
 aux <- unlist(lapply(x, function(x) attr(x, "newCin3Cases")))
 attr(y, "newCin3Cases") <- aux
 aux2 <- unlist(lapply(x, function(x) attr(x, "newSurvivalCases")))
 attr(y, "newSurvivalCases") <- aux2
 aux <- unlist(lapply(x, function(x) attr(x, "md_cost")))
 attr(y, "md_cost") <- aux
 aux <- unlist(lapply(x, function(x) attr(x, "nmd_cost")))
 attr(y, "nmd_cost") <- aux
 aux <- unlist(lapply(x, function(x) attr(x, "i_cost")))
 attr(y, "i_cost") <- aux
 aux <- unlist(lapply(x, function(x) attr(x, "utility")))
 attr(y, "utility") <- aux
 aux <- unlist(lapply(x, function(x) attr(x, "CCdeaths")))
 attr(y, "CCdeaths") <- aux
 aux <- unlist(lapply(x, function(x) attr(x, "Otherdeaths")))
 attr(y, "Otherdeaths") <- aux
```

```
  aux <- unlist(lapply(x, function(x) attr(x, "nCito")))
  attr(y, "nCito") <- aux
  aux <- unlist(lapply(x, function(x) attr(x, "nVPH")))
  attr(y, "nVPH") <- aux
  rownames(y) <- NULL
  y
})
fullDat <- data.frame()
attr(fullDat, "newCases")        <- data.frame()
attr(fullDat, "newCin1Cases")    <- data.frame()
attr(fullDat, "newCin2Cases")    <- data.frame()
attr(fullDat, "newCin3Cases")    <- data.frame()
attr(fullDat, "newSurvivalCases") <- data.frame()
attr(fullDat, "md_cost")         <- data.frame()
attr(fullDat, "nmd_cost")        <- data.frame()
attr(fullDat, "i_cost")          <- data.frame()
attr(fullDat, "utility")         <- data.frame()
attr(fullDat, "CCdeaths")        <- data.frame()
attr(fullDat, "Otherdeaths")     <- data.frame()
attr(fullDat, "nCito")           <- data.frame()
attr(fullDat, "nVPH")            <- data.frame()
for(i in 1:length(listDat)){
  fullDat <- rbind(fullDat, cbind(listDat[[i]], sim=i))
  attr(fullDat, "newCases") <- rbind(attr(fullDat, "newCases"),
                                     attr(listDat[[i]], "newCases"))
  attr(fullDat, "newCin1Cases") <- rbind(attr(fullDat, "newCin1Cases"),
                                         attr(listDat[[i]],"newCin1Cases"))
  attr(fullDat, "newCin2Cases") <- rbind(attr(fullDat, "newCin2Cases"),
                                         attr(listDat[[i]],"newCin2Cases"))
  attr(fullDat, "newCin3Cases") <- rbind(attr(fullDat, "newCin3Cases"),
                                         attr(listDat[[i]],"newCin3Cases"))
  attr(fullDat, "newSurvivalCases") <- rbind(attr(fullDat, "newSurvivalCases"),
                                             attr(listDat[[i]], "newSurvivalCases"))
  attr(fullDat, "md_cost") <- rbind(attr(fullDat, "md_cost"),
                                    attr(listDat[[i]], "md_cost"))
  attr(fullDat, "nmd_cost") <- rbind(attr(fullDat, "nmd_cost"),
                                     attr(listDat[[i]], "nmd_cost"))
  attr(fullDat, "i_cost") <- rbind(attr(fullDat, "i_cost"),
                                   attr(listDat[[i]], "i_cost"))
  attr(fullDat, "utility") <- rbind(attr(fullDat, "utility"),
                                    attr(listDat[[i]], "utility"))
  attr(fullDat, "CCdeaths") <- rbind(attr(fullDat, "CCdeaths"),
                                     attr(listDat[[i]], "CCdeaths"))
  attr(fullDat, "Otherdeaths") <- rbind(attr(fullDat, "Otherdeaths"),
                                        attr(listDat[[i]], "Otherdeaths"))
  attr(fullDat, "nCito") <- rbind(attr(fullDat, "nCito"),
                                  attr(listDat[[i]], "nCito"))
  attr(fullDat, "nVPH") <- rbind(attr(fullDat, "nVPH"),
                                 attr(listDat[[i]], "nVPH"))
}

fullDat$age <- ageGroups[1, 1]:(stopYear-1)
rownames(attr(fullDat, "newCases")) <- paste0("sim", 1:Nsim)
newCases1 <- attr(fullDat, "newCases")
rownames(attr(fullDat, "newCin1Cases")) <- paste0("sim", 1:Nsim)
newCin1Cases1 <- attr(fullDat, "newCin1Cases")
rownames(attr(fullDat, "newCin2Cases")) <- paste0("sim", 1:Nsim)
newCin2Cases1 <- attr(fullDat, "newCin2Cases")
rownames(attr(fullDat, "newCin3Cases")) <- paste0("sim", 1:Nsim)
newCin3Cases1 <- attr(fullDat, "newCin3Cases")
rownames(attr(fullDat, "newSurvivalCases")) <- paste0("sim", 1:Nsim)
newSurvivalCases1 <- attr(fullDat, "newSurvivalCases")
rownames(attr(fullDat, "md_cost")) <- paste0("sim", 1:Nsim)
mdcost1 <- attr(fullDat, "md_cost")
rownames(attr(fullDat, "nmd_cost")) <- paste0("sim", 1:Nsim)
nmdcost1 <- attr(fullDat, "nmd_cost")
rownames(attr(fullDat, "i_cost")) <- paste0("sim", 1:Nsim)
icost1 <- attr(fullDat, "i_cost")
rownames(attr(fullDat, "utility")) <- paste0("sim", 1:Nsim)
utility1 <- attr(fullDat, "utility")
```

```
  rownames(attr(fullDat, "CCdeaths")) <- paste0("sim", 1:Nsim)
  CCdeaths1 <- attr(fullDat, "CCdeaths")
  rownames(attr(fullDat, "Otherdeaths")) <- paste0("sim", 1:Nsim)
  Otherdeaths1 <- attr(fullDat, "Otherdeaths")
  rownames(attr(fullDat, "nCito")) <- paste0("sim", 1:Nsim)
  nCito1 <- attr(fullDat, "nCito")
  rownames(attr(fullDat, "nVPH")) <- paste0("sim", 1:Nsim)
  nVPH1 <- attr(fullDat, "nVPH")
  attr(fullDat, "Call") <- ll
  return(fullDat)
}


### Example validation
library(gdata)
library(mc2d)
library(mco)
library(nloptr)
library(WriteXLS)

source("R/simCohort.R")
source("R/NHvalida.R")
source("R/NHvalida_crs.R")
source("R/eucl.distance.R")

prev.obs <- read.xls("Data/HNObsPrev.xls") ### HPV prevalence
inc.obs  <- read.xls("Data/HNObsInc.xls")  ### CC incidence

### Calibration (including optimization) from a good input matrix
probs <- read.xls("Data/No calibration/probs_nc_good.xls")
system.time(probs_nm <- NHvalida(probs, prev.obs, inc.obs, N=2, keep=2, p.change=0, opt=TRUE))     ### Nelder-Mead algorithm
system.time(probs_rs <- NHvalidacrs(probs, prev.obs, inc.obs, N=2, keep=2, p.change=0, opt=TRUE))  ### Controlled Random Search
algorithm

### Calibration (including optimization) from a bad input matrix
probs <- read.xls("Data/No calibration/probs_nc_bad.xls")
### Nelder-Mead algorithm
system.time(probs_nm <- NHvalida(probs, prev.obs, inc.obs, N=2, keep=2, p.change=0, opt=TRUE))
### Controlled Random Search algorithm
system.time(probs_rs <- NHvalidacrs(probs, prev.obs, inc.obs, N=2, keep=2, p.change=0, opt=TRUE))
```

R code to reproduce the figures

```
### figure 1
library(ggplot2)
library(gdata)
library(gridExtra)

source("R/byAgeGroup.R")
source("R/simCohort.R")
source("R/plotPrevalence.R")
source("R/plotIncidence.R")

prev.obs   <- read.xls("Data/HNObsPrev.xls") ### HPV prevalence
inc.obs    <- read.xls("Data/HNObsInc.xls")  ### CC incidence
probs.nm.g <- read.xls("Data/NM calibration/probs_nm_good.xls")     ### Nelder-Mead calibrated matrix
probs.nm.b <- read.xls("Data/NM calibration/probs_nm_bad.xls")      ### Nelder-Mead calibrated matrix
probs.nc.g <- read.xls("Data/No calibration/probs_nc_good.xls")     ### Non calibrated matrix (good)
probs.nc.b <- read.xls("Data/No calibration/probs_nc_bad.xls")      ### Non calibrated matrix (bad)
probs.rs.g <- read.xls("Data/CRS calibration/probs_rs_good.xls")    ### Nelder-Mead calibrated matrix
probs.rs.b <- read.xls("Data/CRS calibration/probs_rs_bad.xls")     ### Nelder-Mead calibrated matrix
probs.mc   <- read.xls("Data/Manual calibration/probs_mc.xls")      ### Manually calibrated matrix

res.nc.g <- simCohort(probs1=probs.nc.g, Nsim = 100,
              figoSymProb = c(0.11, 0.23, 0.66, 0.9), ### Probability to develop symptoms in FIGO states
              vaccinePrice.md = 0, vaccinePrice.nmd = 0, vaccinePrice.i = 0, screenPrice.md = 0, screenPrice.nmd = 0, screenPrice.i = 0,
              costCoefs.md = c(0, 0, 248.2, 1461.0, 1461.0, 5417.3, 12136.3, 22583.6, 33222.5, 0, 0, 0),
              costCoefs.nmd = c(0, 0, 79.5, 189.6, 189.6, 214.0, 214.0, 214.0, 214.0, 0, 0, 0),
              costCoefs.i = c(0, 0, 0, 0, 0, 0, 0, 0, 0, 0, 0, 0),
```

```
            vaccineCov = 0, utilityCoefs = c(1, 1, 0.987, 0.87, 0.87, 0.76, 0.67, 0.67, 0.67, 0.938, 0, 0),
            screenProbs = c(0,0,1,1,1,0.9688,0.9066,0.7064,0.3986,0,0,0), ndoses = 0)

res.nc.b <- simCohort(probs1=probs.nc.b, Nsim = 100,
             figoSymProb = c(0.11, 0.23, 0.66, 0.9), ### Probability to develop symptoms in FIGO states
             vaccinePrice.md = 0, vaccinePrice.nmd = 0, vaccinePrice.i = 0, screenPrice.md = 0, screenPrice.nmd = 0, screenPrice.i = 0,
             costCoefs.md = c(0, 0, 248.2, 1461.0, 1461.0, 5417.3, 12136.3, 22583.6, 33222.5, 0, 0, 0),
             costCoefs.nmd = c(0, 0, 79.5, 189.6, 189.6, 214.0, 214.0, 214.0, 214.0, 0, 0, 0),
             costCoefs.i = c(0, 0, 0, 0, 0, 0, 0, 0, 0, 0, 0, 0),
             vaccineCov = 0, utilityCoefs = c(1, 1, 0.987, 0.87, 0.87, 0.76, 0.67, 0.67, 0.67, 0.938, 0, 0),
             screenProbs = c(0,0,1,1,1,0.9688,0.9066,0.7064,0.3986,0,0,0), ndoses = 0)

res.mc <- simCohort(probs1=probs.mc, Nsim = 100,
            figoSymProb = c(0.11, 0.23, 0.66, 0.9), ### Probability to develop symptoms in FIGO states
            vaccinePrice.md = 0, vaccinePrice.nmd = 0, vaccinePrice.i = 0, screenPrice.md = 0, screenPrice.nmd = 0, screenPrice.i = 0,
            costCoefs.md = c(0, 0, 248.2, 1461.0, 1461.0, 5417.3, 12136.3, 22583.6, 33222.5, 0, 0, 0),
            costCoefs.nmd = c(0, 0, 79.5, 189.6, 189.6, 214.0, 214.0, 214.0, 214.0, 0, 0, 0),
            costCoefs.i = c(0, 0, 0, 0, 0, 0, 0, 0, 0, 0, 0, 0),
            vaccineCov = 0, utilityCoefs = c(1, 1, 0.987, 0.87, 0.87, 0.76, 0.67, 0.67, 0.67, 0.938, 0, 0),
            screenProbs = c(0,0,1,1,1,0.9688,0.9066,0.7064,0.3986,0,0,0), ndoses = 0)

res.nm.g <- simCohort(probs1=probs.nm.g, Nsim = 100,
           figoSymProb = c(0.11, 0.23, 0.66, 0.9), ### Probability to develop symptoms in FIGO states
           vaccinePrice.md = 0, vaccinePrice.nmd = 0, vaccinePrice.i = 0, screenPrice.md = 0, screenPrice.nmd = 0, screenPrice.i = 0,
           costCoefs.md = c(0, 0, 248.2, 1461.0, 1461.0, 5417.3, 12136.3, 22583.6, 33222.5, 0, 0, 0),
           costCoefs.nmd = c(0, 0, 79.5, 189.6, 189.6, 214.0, 214.0, 214.0, 214.0, 0, 0, 0),
           costCoefs.i = c(0, 0, 0, 0, 0, 0, 0, 0, 0, 0, 0, 0),
           vaccineCov = 0, utilityCoefs = c(1, 1, 0.987, 0.87, 0.87, 0.76, 0.67, 0.67, 0.67, 0.938, 0, 0),
           screenProbs = c(0,0,1,1,1,0.9688,0.9066,0.7064,0.3986,0,0,0), ndoses = 0)

res.nm.b <- simCohort(probs1=probs.nm.b, Nsim = 100,
             figoSymProb = c(0.11, 0.23, 0.66, 0.9), ### Probability to develop symptoms in FIGO states
             vaccinePrice.md = 0, vaccinePrice.nmd = 0, vaccinePrice.i = 0, screenPrice.md = 0, screenPrice.nmd = 0, screenPrice.i = 0,
             costCoefs.md = c(0, 0, 248.2, 1461.0, 1461.0, 5417.3, 12136.3, 22583.6, 33222.5, 0, 0, 0),
             costCoefs.nmd = c(0, 0, 79.5, 189.6, 189.6, 214.0, 214.0, 214.0, 214.0, 0, 0, 0),
             costCoefs.i = c(0, 0, 0, 0, 0, 0, 0, 0, 0, 0, 0, 0),
             vaccineCov = 0, utilityCoefs = c(1, 1, 0.987, 0.87, 0.87, 0.76, 0.67, 0.67, 0.67, 0.938, 0, 0),
             screenProbs = c(0,0,1,1,1,0.9688,0.9066,0.7064,0.3986,0,0,0), ndoses = 0)

res.rs.g <- simCohort(probs1=probs.rs.g, Nsim = 100,
             figoSymProb = c(0.11, 0.23, 0.66, 0.9), ### Probability to develop symptoms in FIGO states
             vaccinePrice.md = 0, vaccinePrice.nmd = 0, vaccinePrice.i = 0, screenPrice.md = 0, screenPrice.nmd = 0, screenPrice.i = 0,
             costCoefs.md = c(0, 0, 248.2, 1461.0, 1461.0, 5417.3, 12136.3, 22583.6, 33222.5, 0, 0, 0),
             costCoefs.nmd = c(0, 0, 79.5, 189.6, 189.6, 214.0, 214.0, 214.0, 214.0, 0, 0, 0),
             costCoefs.i = c(0, 0, 0, 0, 0, 0, 0, 0, 0, 0, 0, 0),
             vaccineCov = 0, utilityCoefs = c(1, 1, 0.987, 0.87, 0.87, 0.76, 0.67, 0.67, 0.67, 0.938, 0, 0),
             screenProbs = c(0,0,1,1,1,0.9688,0.9066,0.7064,0.3986,0,0,0), ndoses = 0)

res.rs.b <- simCohort(probs1=probs.rs.b, Nsim = 100,
             figoSymProb = c(0.11, 0.23, 0.66, 0.9), ### Probability to develop symptoms in FIGO states
             vaccinePrice.md = 0, vaccinePrice.nmd = 0, vaccinePrice.i = 0, screenPrice.md = 0, screenPrice.nmd = 0, screenPrice.i = 0,
             costCoefs.md = c(0, 0, 248.2, 1461.0, 1461.0, 5417.3, 12136.3, 22583.6, 33222.5, 0, 0, 0),
             costCoefs.nmd = c(0, 0, 79.5, 189.6, 189.6, 214.0, 214.0, 214.0, 214.0, 0, 0, 0),
             costCoefs.i = c(0, 0, 0, 0, 0, 0, 0, 0, 0, 0, 0, 0),
             vaccineCov = 0, utilityCoefs = c(1, 1, 0.987, 0.87, 0.87, 0.76, 0.67, 0.67, 0.67, 0.938, 0, 0),
             screenProbs = c(0,0,1,1,1,0.9688,0.9066,0.7064,0.3986,0,0,0), ndoses = 0)

res.nc.g.hpv <- byAgeGroup(plotPrevalence(res.nc.g))[, 2]
res.nc.g.cc  <- byAgeGroup(plotIncidence(res.nc.g)[[2]])[, 2]
res.nc.b.hpv <- byAgeGroup(plotPrevalence(res.nc.b))[, 2]
res.nc.b.cc  <- byAgeGroup(plotIncidence(res.nc.b)[[2]])[, 2]
res.mc.hpv   <- byAgeGroup(plotPrevalence(res.mc))[, 2]
res.mc.cc    <- byAgeGroup(plotIncidence(res.mc)[[2]])[, 2]
res.nm.g.hpv <- byAgeGroup(plotPrevalence(res.nm.g))[, 2]
res.nm.g.cc  <- byAgeGroup(plotIncidence(res.nm.g)[[2]])[, 2]
res.nm.b.hpv <- byAgeGroup(plotPrevalence(res.nm.b))[, 2]
res.nm.b.cc  <- byAgeGroup(plotIncidence(res.nm.b)[[2]])[, 2]
res.rs.g.hpv <- byAgeGroup(plotPrevalence(res.rs.g))[, 2]
res.rs.g.cc  <- byAgeGroup(plotIncidence(res.rs.g)[[2]])[, 2]
res.rs.b.hpv <- byAgeGroup(plotPrevalence(res.rs.b))[, 2]
```

```
res.rs.b.cc  <- byAgeGroup(plotIncidence(res.rs.b)[[2]])[, 2]

graph <- data.frame(HPV=c(prev.obs[, 2], res.nc.b.hpv, res.nc.g.hpv, res.mc.hpv, res.nm.b.hpv, res.nm.g.hpv, res.rs.b.hpv, res.rs.g.hpv),
             CC=c(inc.obs[, 2], res.nc.b.cc, res.nc.g.cc, res.mc.cc, res.nm.b.cc, res.nm.g.cc, res.rs.b.cc, res.rs.g.cc),
             Age=rep(c("10-14", "15-19", "20-24", "25-29", "30-34", "35-39", "40-44", "45-49", "50-54", "55-59", "60-64",
                  "65-69", "70-74", "75-79", "80-84"), 8),
             Calibration=c(rep("Observed", 15), rep("NC (bad)", 15), rep("NC (good)", 15),
                  rep("MC", 15), rep("NM (bad)", 15), rep("NM (good)", 15),
                  rep("CRS (bad)", 15), rep("CRS (good)", 15)))

graph$Calibration <- factor(graph$Calibration, unique(graph$Calibration)[c(1,2,3,4,5,6,7,8)])

hpv <- ggplot(graph, aes(x=Age, y=HPV, group=Calibration, color=Calibration)) + geom_line(aes(linetype=Calibration))+
  geom_point(aes(shape=Calibration, size=1.3)) +
  ylab ('') + ggtitle("HPV prevalence") + theme(plot.title = element_text(hjust = 0.5)) + xlab('') +
  scale_linetype_manual(values = c(1,2,3,4,5,6,1,2)) + scale_shape_manual(values=c(0,1,2,3,4,5,6,7)) +
  theme(text = element_text(size=11, face="bold"), legend.position="none")+scale_color_manual(values=c(2,1,1,1,1,1,1,1))
cc <- ggplot(graph, aes(x=Age, y=CC, group=Calibration, color=Calibration)) +
  geom_line(aes(linetype=Calibration)) +
  geom_point(aes(shape=Calibration, size=1.3)) +
  ylab ('') + ggtitle("Cervical cancer incidence (x 100,000 women)") + theme(plot.title = element_text(hjust = 0.5)) +
  scale_linetype_manual(values = c(1,2,3,4,5,6,1,2)) + scale_shape_manual(values=c(0,1,2,3,4,5,6,7)) +
  theme(text = element_text(size=11, face="bold"), legend.position="bottom", legend.title=element_text(size=12, face="bold"),
legend.text=element_text(size = 12, face = "bold")) +
  guides(size=FALSE)+scale_color_manual(values=c(2,1,1,1,1,1,1,1))
postscript("Article/fig1.eps", width = 1074, height = 586)
  grid.arrange(hpv, cc, ncol=1)
dev.off()

### figure 2
library(gdata)
library(ggplot2)

pchange <- read.xls("Data/percentage_results.xls")
pchange$Calibration <- factor(pchange$Calibration, unique(pchange$Calibration)[c(1,2,3,4,5,6,7,8)])
colnames(pchange)[2]<-"Strategy"
pchange$Strategy <- factor(pchange$Strategy, unique(pchange$Strategy)[c(1,2,5,3,4,8,6,7)])
dat_hlines <- data.frame(Calibration=levels(pchange$Calibration), hline=c(0.168, 0.087, 0.041, 0.046, 0.035, 0.033))

postscript("Article/pchange.eps", width = 1074, height = 586)
  ggplot(pchange, aes(Calibration, change))+geom_point(aes(shape = Strategy, color=Calibration, size=1.3)) + ylab("Percent change of
CERs")+
    scale_shape_manual(values=c(0,1,2,3,4,5,6,7))+xlab("")+scale_y_continuous(labels = scales::percent)+
    annotate("text", x = 1:6, y = c(0.125,0.075,0.074,0.064,0.076,0.065), label = c("12.5%", "7.5%", "7.4%", "6.4%", "7.6%",
"6.5%"))+guides(color=FALSE)+
    guides(size=FALSE)+geom_segment(aes(x = 0.75, y = 0.168, xend = 1.25, yend = 0.168))+geom_segment(aes(x = 1.75, y = 0.087, xend =
2.25, yend = 0.087))+
    geom_segment(aes(x = 2.75, y = 0.041, xend = 3.25, yend = 0.041))+geom_segment(aes(x = 3.75, y = 0.046, xend = 4.25, yend = 0.046))+
    geom_segment(aes(x = 4.75, y = 0.035, xend = 5.25, yend = 0.035))+geom_segment(aes(x = 5.75, y = 0.033, xend = 6.25, yend = 0.033))+
    scale_color_manual(values=c(1,2,3,4,5,6))+theme(text = element_text(size=11, face="bold"), legend.title=element_text(size=12,
face="bold"), legend.text=element_text(size = 12, face = "bold"))
dev.off()

### figure 3
library(gdata)
library(ggplot2)
library(scales)

icers <- read.xls("ICERs.xlsx")
icers$Calibration <- factor(icers$Calibration, unique(icers$Calibration)[c(7,1,2,3,4,5,6)])
icers$Strategy <- factor(icers$Strategy, unique(icers$Strategy)[c(1,4,2,3,7,5,6)])

postscript("Article/icers.eps", width = 1074, height = 586)
  ggplot(icers, aes(x=Calibration, y=ICER, group=Calibration)) + geom_point(aes(shape = Strategy, color=Calibration, size=1.3)) +
  ylab ("ICER (€/QALY)") + ggtitle("") + theme(plot.title = element_text(hjust = 0.5)) + xlab("")+
    scale_color_manual(values=c("orange",1,2,3,4,5,6))+scale_y_continuous(breaks=seq(1000, 15000, 1000), labels=comma)+
    scale_shape_manual(values=c(1,2,3,4,5,6,7,8))+guides(color=FALSE, size=FALSE)+
    theme(text = element_text(size=11, face="bold"), legend.title=element_text(size=12, face="bold"), legend.text=element_text(size = 12,
face = "bold"))
dev.off()
```
